# Pholidota‐Inspired Electronic Skin Possessing Terahertz‐Wave Reflection–Absorption–Transmission Switchability

**DOI:** 10.1002/advs.202518194

**Published:** 2026-06-03

**Authors:** Shangjing Li, Jiangsong Hou, Yifeng Ruan, Jiang Du, Jun Qiu

**Affiliations:** ^1^ School of Materials Science and Engineering Tongji University Shanghai P. R. China; ^2^ Key Laboratory of Textile Fiber and Products, Ministry of Education Wuhan Textile University Wuhan China; ^3^ School of Materials Science and Engineering and Key Laboratory of Advanced Civil Engineering Materials of Education of Ministry Shanghai P. R. China

**Keywords:** biomimetic material, electronic skin, strain regulation, terahertz shielding, tunable terahertz‐wave absorption

## Abstract

The escalating complexity of electromagnetic environments demands intelligent materials capable of real‐time adaptive electromagnetic responses to reconcile conflicting requirements for stealth, transmission, and shielding. Current electromagnetic protection systems face two bottlenecks: static material characteristics limit the active switching of reflection (R), absorption (A), and transmission (T) states, and the contradiction among the three hinders their dynamic coexistence. To address this challenge, a self‐adaptive reflection–absorption–transmission switchable electronic skin (RATS‐E‐skin) was designed to harmonize perception with terahertz‐wave switchability. Inspired by pholidota, the RATS‐E‐skin combines flow‐deformable liquid metal, graphene sheets, and iron oxide nanorods into a ternary architecture. This system overcomes the intrinsic incompatibility among RAT mechanisms through strain‐mediated microstructural reconfiguration, enabling the four‐state reversible switching of terahertz‐wave reflection (32.4 dB shielding), absorption (60.2 dB reflection loss), transmission (up to 76.7%), and secondary reflection (50.8 dB shielding). Critically, the material achieves multimodal sensing with resistance sensitivity and cyclic stability, and in response to external demands, it issues deformation instructions, verifies the deformation state after the equipment executes the action, and thus forms a closed‐loop adaptation to precisely switch the corresponding electromagnetic functions. This bioinspired approach shows potential for deformable equipment armor and multifunctional wearable systems in both civilian and defense applications.

## Graphical abstract

The pholidota‐inspired electronic skin is successfully constructed, originally realized four‐state reversible switching of terahertz‐wave reflection, absorption, transmission, and secondary reflection. In response to external demands, it issues deformation instructions, verifies the deformation state after the equipment executes the action, and thus forms a closed‐loop adaptation to precisely switch the corresponding electromagnetic functions.

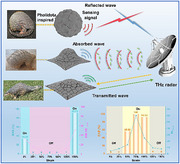

## 1 Introduction

The rapid advancement of communication technologies and flexible electronics has intensified the demand for electromagnetic functional materials capable of transitioning from static single state operation to intelligent dynamic adaptation. Current electromagnetic materials face two fundamental limitations. First, their static properties confine them to fixed functions (reflection/shielding, absorption/stealth, or transmission/communication), precluding real‐time switching among these states via active regulation. Second, inherent contradictions exist in the reflection‐absorption‐transmission (RAT) mechanisms: high reflection requires continuous conductive networks, high absorption relies on discrete loss units, and high transmission demands minimal reflection and absorption. These constraints impede the dynamic balancing of signal transmission and electromagnetic protection in increasingly complex environments. This operational‐protection dichotomy manifests acutely in critical applications. Unmanned aerial vehicle (UAV) requires instantaneous switching between absorption (stealth), transmission (communication), and reflection (anti‐jamming). Similarly, existing protective materials compromise the functionality of electromagnetic transceiver antennas or radomes in communication equipment, exemplified by the vulnerable external placement of antennas on some civil aircraft, which exposes them to safety hazards. This persistent incompatibility underscores the urgent need for active RAT‐switchable materials [1, 2, 3, 4, 5, 6, 7]. Addressing this challenge requires not only tunable electromagnetic properties but also integrated environmental perception. Electronic skin (E‐skin) technology, crucial for safety monitoring, enables such perception through characteristic changes in resistance to achieve quantitative identification of stimulus signals, providing the sensory input required to drive structural adjustments within the material and ultimately facilitate active regulation of electromagnetic functions [8, 9, 10, 11]. Such a “sensing‐response” functionality promises to advance electromagnetic stealth from fixed mode to truly adaptive systems. However, to the best of our knowledge, intelligent materials integrating perception, defense, stealth, and communication functions, especially in the terahertz band, likely for next‐generation 6G’ networks, have not been reported.

Specifically, current electromagnetic protection systems are constrained by static material properties and lack the capacity to actively regulate wave—material interactions. An effective strategy is to regulate the material's electromagnetic parameters via external stimuli, such as stress, temperature, [12] light, voltage, [13] and magnetic field, so as to achieve dynamic regulation of electromagnetic responses. Among these stimuli, both mechanically‐reconfiguration and electronically‐reconfiguration are common methods [14]. The former features flexibility and conformability, while the latter is more suitable for scenarios requiring high‐precision cycling. Recently, many studies have used these stimulus‐driven regulation strategies to prepare intelligent materials, such as poly(3,4‐ethylenedioxythiophene) polystyrene sulfonate (PEDOT:PSS)/cellulose, poly (*N*‐isopropylacrylamide) (PNIPAM)/PEDOT:PSS/MXene and reduced graphene oxide (rGO)/VO_2_ aerogels; [15, 16, 17] a functional carbon spring; [18] a bionic octopus composite foam; [19] graphene oxide–quercetin (GO/QC), [20] polyaniline–sodium alginate (PANI/SA), [21] PNIPAM/MXene/carbon nanotubes/polyvinyl alcohol (PMCP), [22] and MXene‐based (M)–organic hydrogels; [23] a magnetic liquid metal (LM) film; [24] and an rGO/polymer elastomer, [25] possessing “on/off” mode stealth or shielding. However, the inherent contradiction among RAT mechanisms (where absorptivity *A(ω)*, transmittance *T(ω)*, and reflectivity *R(ω)* must satisfy *A(ω)* + *T(ω)* + *R(ω)* = 1) makes it difficult for a single material to be compatible with all three simultaneously [26]. High‐reflectivity materials require the formation of conductive pathways. High‐absorption materials require the dispersion of loss‐generating components in the matrix instead of the formation of a continuous conductive network, while high‐transmittance materials require weak wave absorption and reflection for waves to pass through them. This mechanism conflict, coupled with the need for coordinated regulation of sensing functions and electromagnetic properties, renders achieving this closed‐loop response a great challenge, as traditional electromagnetic materials lack intrinsic sensing capabilities, while conventional E‐skin lacks integrated electromagnetic functional regulation.

Bionics has emerged as a pivotal driver for advanced material innovation, particularly in electromagnetics and sensing technologies [27, 28, 29, 30]. Natural organisms offer optimized solution for multifunctional systems, with the scale‐driven mechanism of pholidota being a typical example. Through its three‐step response of threat perception, self‐deformation, and function switching, it achieves adaptive feedback to the external environment. After capturing external threat signals via its own sensory system, pholidota drives the deformation of its scales through muscle contraction and relaxation, which correlates with distinct functions and environmental responses. An expanded posture facilitates environmental interaction (“communicating” analogous to electromagnetic wave transmission), semi‐curled posture enables environmental blending (analogous to stealth via absorption), and a fully curled state provides mechanical defense (impact reflection akin to electromagnetic shielding). This complete system composed of perception, actuation, and function execution units serves as the foundation for its dynamic response, while resisting, evading or interacting with external energy through structural deformation constitutes the core regulatory mechanism of this biological system.

Herein, a self‐adaptive RAT‐switchable E‐skin was developed. The adaptive regulatory mechanism of pholidota scales in responding to mechanical energy is transposed across fields to the material design for electromagnetic energy regulation. Given that mechanical energy and electromagnetic energy are inherently categorized as energy in essence, an electromagnetic energy regulation system corresponding to the steps of biological systems is thus constructed, enabling the dynamic adaptive response of perception‐deformation‐function switching in the terahertz band: detection meters identify electromagnetic signals, which then triggers the active shape change of deformable equipment to drive the E‐skin deformation. Consequently, the unstretched E‐skin achieves terahertz‐wave reflection via dense conductive networks, corresponding to the defensive state of pholidota with fully interlocked scales. While stretching triggers absorption‐transmission switching through strain‐mediated dynamic reconstruction of microstructures, namely the redistribution of graphene sheet and iron oxide (Fe_2_O_3_) nanorods and the coordinated LM deformation, matching the semi‐curled and fully extended scale states of pholidota, respectively. This bioinspired architecture overcomes the fundamental RAT mechanism incompatibility, enabling electromagnetic function switching validated in deformable armor and tunable stealth surfaces.

## 2 Results and Discussion

### 2.1 Design of Bionic Pholidota‐Inspired Structure

**FIGURE 1:**
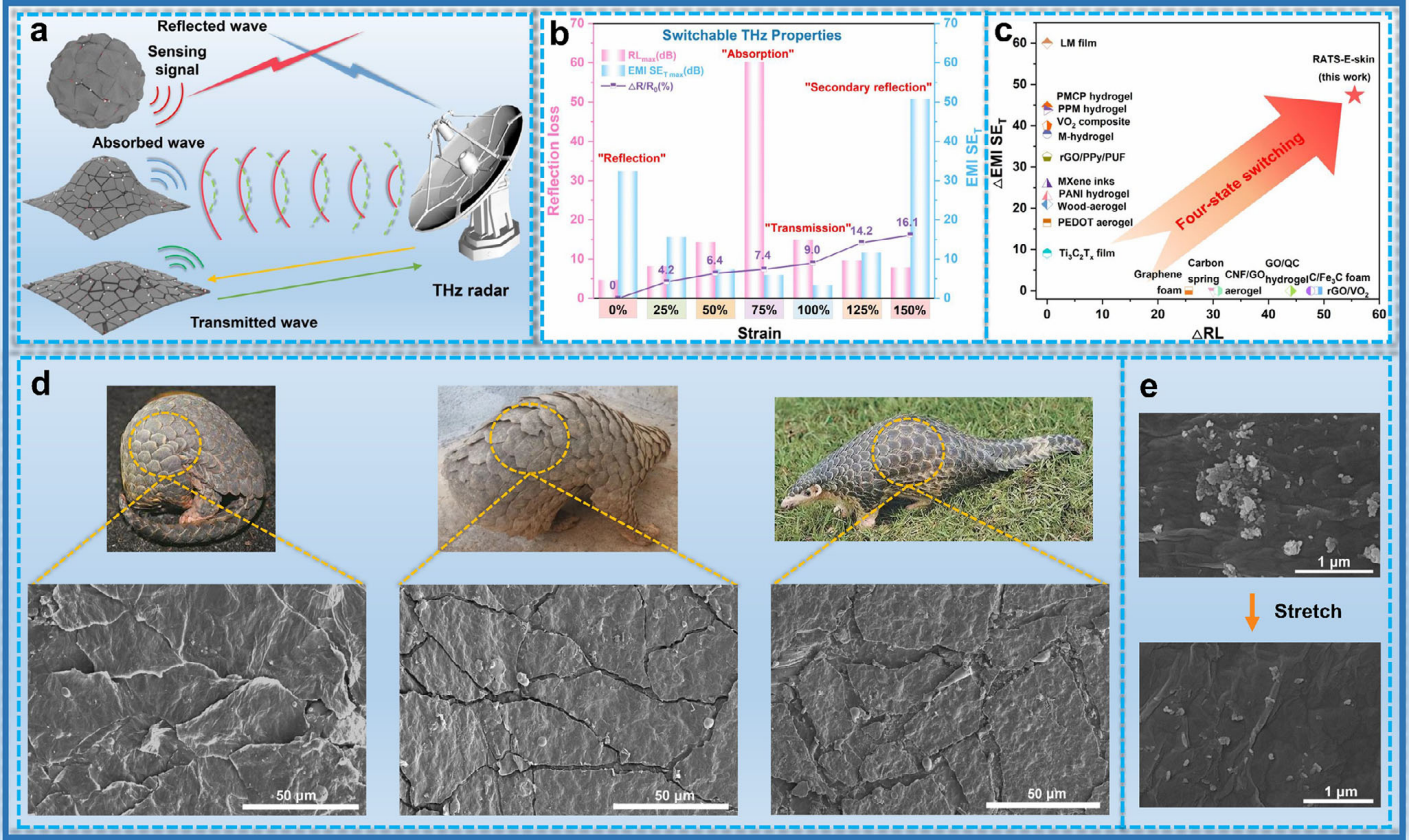
Applications of the RATS‐E‐skin, possessing switchable terahertz‐wave properties, and the design of the pholidota‐inspired structure. (a) RAT‐E‐skin's active closed‐loop mechanism featuring strain‐adaptive deformation in response to external demands. (b) Correlation curves between relative resistance changes and terahertz performance of the RATS‐E‐skin at 0%–150% strain. (c) Comparison of the RATS‐E‐skin's maximum reflection loss change (△RL) and shielding effectiveness change (△EMI SE_T_) with electromagnetically regulated materials in the open literature. (d) SEM images of the RATS‐E‐skin possessing the bionic pholidota‐inspired structure. The fully curled, semi‐curled and expanded postures of pholidota scales correspond to the RATS‐E‐skin at tensile strains of 0%, 75%, and 100%. (e) SEM images of Fe_2_O_3_ nanorods in the RATS‐E‐skin changing from clustered to independent distributions during stretching, respectively.

Inspired by the functional response mechanism of pholidota, this work develops a biomimetic design for reconfigurable electromagnetic materials. The fabricated RATS‐E‐skin integrates graphene sheets, flow‐deformable LM, and Fe_2_O_3_ nanorods into a ternary architecture. This integration enables it, after identifying external electromagnetic signals by relying on the detector built into the equipment, to determine the target strain and issue deformation instructions based on its strain‐dependent terahertz characteristics. The micro‐drive unit then drives the equipment to deform and in turn induces its own deformation. Meanwhile, it real‐time senses the deformation degree and verifies whether the target state is achieved, ultimately realizing the precise switching of electromagnetic functions. Crucially, the strain‐mediated microstructural reconfiguration, achieved through graphene sheet sliding, LM droplet elongation, and Fe_2_O_3_ nanorod dispersion, drives broadband terahertz‐wave switching among four states: reflection (shielding)—absorption (stealth)—transmission—secondary reflection (secondary shielding) (Figure 1a,b). Unlike existing materials limited to single‐function optimization (e.g. stealth or shielding), [31, 32, 33, 34, 35, 36] the RATS‐E‐skin simultaneously regulates reflection loss (RL) and electromagnetic interference shielding effectiveness (EMI SE_T_) over dynamic ranges, achieving 55.52 and 47.39 dB at optimal conditions (Figure 1c).

The strain‐responsive electromagnetic switching of the RATS‐E‐skin originates from coordinated microstructural evolution during deformation, characterized by three processes (Figure 1d), sliding‐induced separation of overlapping graphene sheets, LM droplet‐to‐wire morphological transition, and dispersion of clustered Fe_2_O_3_ nanorods into isolated units. In the unstretched state (0% strain), graphene sheets and Fe_2_O_3_ nanorods stack tightly, with LM droplets bridging inter‐sheet junctions to form a highly conductive reflective layer. This ternary synergy yields high electrical conductivity (65.93 S/m) and reflection‐dominated electromagnetic response, matching the functional effect of mechanical defense formed by the stacked scales when pholidota curls up into a ball, both achieving the direct blocking of external energy. At 75% tensile strain, graphene sheets slide laterally, reducing overlap areas and generating microcracks. LM droplets partially fill small cracks but fail to bridge wider gaps (>3 µm). These changes disrupt conductive networks, decreasing conductivity to semiconductor‐level (∼0.452 S/m). Concurrently, interfacial polarization at rGO/LM/Fe_2_O_3_ heterojunctions, coupled with multi‐scattering within expanding gaps, drives the transition to absorption dominance, achieving a stealth effect that corresponds to the camouflage function of environmental integration when pholidota scales are half‐curled, both realizing the evasion and attenuation of external energy. At 100% strain, inter‐sheet separation exceeds LM's bridging capacity. Fe_2_O_3_ nanorods isolation reduces polarization sites (Figure 1e), while material thinning diminishes overall loss. These collectively enhance transmittance, realizing an electromagnetic communication function that corresponds to the environmental interaction function when pholidota scales are fully extended, both achieving the exchange of energy and information with the outside world. In summary, both achieve targeted responses to external energy by virtue of structural deformation differences, which reflects the homology of the regulatory mechanisms for mechanical energy and electromagnetic energy. Whereas when strain reaches 150%, critical stress fractures LM oxide layers, enabling liquid core extrusion that forms metallic wires reconnecting graphene sheets. This reconstructs conductive networks, sharply increasing electrical conductivity and restoring reflection‐dominated behavior. This state is a supplementary protective design of the material for extreme working conditions, which resists extremely intense electromagnetic attacks through the reinforced reflection mode and serves as an extreme protection scenario exclusive to the equipment. In contrast, the 0%–100% strain range is the conventional working interval of the material, within which LM oxide layers remain intact and undergo controllable morphological changes, enabling the stable switching of the three core functions of reflection, absorption, and transmission. Combined with the extreme protection characteristic at 150% strain, the material achieves dynamic and full‐range regulation of electromagnetic responses across 0%–150% strain.

**FIGURE 2:**
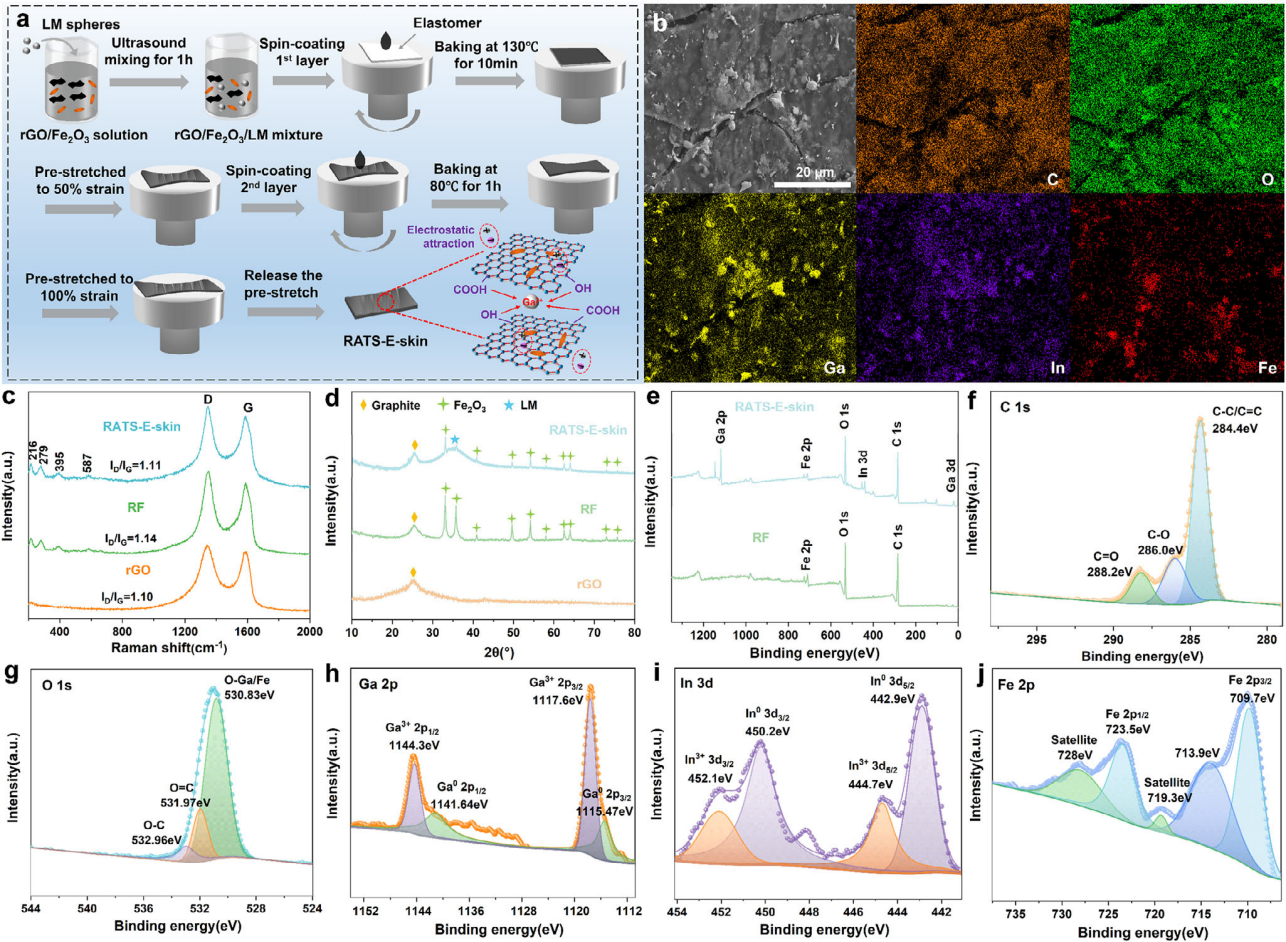
Preparation and characterization of the RATS‐E‐skin. (a) Schematic of the procedures for fabricating the RATS‐E‐skin possessing an overlapping scale structure. (b) EDS mapping of the RATS‐E‐skin. (c) Raman spectra and (d) XRD patterns of the rGO, rGO/Fe_2_O_3_ film (RF), and RATS‐E‐skin. (e) XPS spectra of the RF and RATS‐E‐skin, and high‐resolution (f) C 1*s*, (g) O 1*s*, (h) Ga 2*p*, (i) In 3*d*, and (j) Fe 2*p* spectra of the same.

The schematic of the RATS‐E‐skin's preparation method is shown in Figure 2a. A unique overlapping scale structure is prepared via prestretching and multistep spin‐coating. First, prestretching arranged the graphene sheets in an orderly manner. Then, guided by the prestretching‐generated stress, spin‐coating predominantly and directionally attached the graphene sheets to the substrate surface along the stretching direction, forming a preliminary single‐layer sheet structure. Subsequently, restretching further widened the gaps between the graphene sheets, producing an area overlapping the first layer. Finally, by releasing the prestretching, an overlapping double‐layer scale structure is prepared. Among the various components, the electrostatic interaction between the carboxyl and hydroxyl groups attached to the rGO structure and the LM's Ga^3+^ promoted the LM's close adhesion to the graphene sheets. Simultaneously, the electrostatic attraction between the Fe_2_O_3_ nanorods’ electropositive Fe and rGO oxygen‐containing groups’ electronegative O atoms generated a strong binding force, enabling the three components to construct a multidimensional composite material possessing a stable 1/2/3D structure.

The elemental composition and distribution of the E‐skin were analyzed using energy‐dispersive spectroscopy (EDS) (Figure 2b and Figure S1). C and O were mainly derived from rGO and Fe_2_O_3_, Ga and In were both derived from LM, and Fe was derived from the Fe_2_O_3_ nanorods. According to the elemental distribution, most of LM was clearly distributed between the graphene sheets, connecting them to a conductive structure (Figure S2), and the Fe_2_O_3_ nanorods were homogeneously distributed across the graphene sheets [37]. Raman spectroscopy was employed to analyze the degree of structural defects in the carbon material. As shown in Figure 2c, two typical strong peaks, corresponding to the D and G bands, appeared near 1348 and 1590 cm^−1^, respectively. The D and G bands indicate the degree of carbon atom disorder or the defect extent in the graphite phase and are associated with carbon atoms’ ordered oscillation, respectively. Compared with spectrum of the pure rGO, those of the RF and RATS‐E‐skin exhibit peaks at 216, 279, 395, and 587 cm^−1^, which are attributed to Fe_2_O_3_ [38]. The peak intensity ratio (I_D_/I_G_) indicates the defect density or graphitization degree in the graphite structure. Specifically, for rGO, I_D_/I_G_ = 1.10. For the RF and RATS‐E‐skin, I_D_/I_G_ = 1.14 and 1.11, respectively. The RF's I_D_/I_G_ was higher than that of the rGO, indicating that the formation of the Fe_2_O_3_ nanorods stacked the graphene sheets in a disorderly manner. The RATS‐E‐skin's I_D_/I_G_ value was relatively lower than that when only Fe_2_O_3_ was added, probably because the LM filled some of the gaps or defects between the Fe_2_O_3_ and graphene sheets, reducing the defect exposure degree. X‐ray diffraction (XRD) was utilized to identify the crystalline phases in the samples (Figure 2d). The XRD pattern of the pure rGO showed an obvious, relatively broad characteristic peak at 25°, corresponding to the graphite (002) crystal plane. In the RF's XRD pattern, multiple diffraction peaks appeared at 33.08°, 35.78°, 40.86°, 54.17°, 58.01°, 63.99°, and 75.67°, corresponding to the Fe_2_O_3_ (104), (110), (113), (116), (018), (300), and (220) planes, respectively (JCPDS No.33‐0664). In the RATS‐E‐skin's XRD pattern, the broad characteristic peak near 35° was attributed to the amorphous gallium–indium alloy [39]. X‐ray photoelectron spectroscopy (XPS) was employed to determine the elemental valence and chemical bonding states. The full‐spectrum analysis revealed that the RATS‐E‐skin contained C, O, Ga, In, and Fe (Figure 2e). The high‐resolution spectrum of C1*s* (Figure 2f), exhibited characteristic peaks at 284.4, 286.0, and 288.2 eV, corresponding to C–C/C═C, C–O, and C═O bonds, respectively. In the O 1*s* spectrum, the peaks at 530.83, 531.97, and 532.96 eV corresponded to O–Ga/Fe, O═C, and O–C bonds, respectively (Figure 2g), suggesting that a few residual oxygen‐containing functional groups remained after the graphene oxide was reduced and that they strongly bonded with the LM and Fe_2_O_3_ nanorods. The characteristic peaks in the Ga 2*p* spectrum corresponded to two gallium chemical states. The high‐intensity peaks centered at 1117.6 and 1144.3 eV were ascribed to Ga^3+^ 2*p*_3/2_ and 2*p*_1/2_. In contrast, the weak peaks at 1115.47 and 1141.64 eV corresponded to metallic Ga's 2*p*_3/2_ and 2*p*_1/2_, indicating that the Ga was mainly derived from the oxide layer (Ga_2_O_3_) of the LM and was oxidized (Figure 2h). The characteristic peaks at 442.9 and 450.2 eV were attributed to metallic In's 3*d*_5/2_ and 3*d*_3/2_, and the peaks at 444.7 and 452.1 eV were assigned to In^3+^ 3*d*_5/2_ and 3*d*_3/2_, indicating that the indium was mainly metallic and, therefore, possessed relatively stable chemical properties at room temperature (Figure 2i) [40, 41]. The Fe2*p* spectrum exhibited characteristic peaks at 709.7, 713.9, and 723.5 eV, which were, respectively, assigned to Fe 2*p*_3/2_ and 2*p*_1/2_, and were accompanied by satellite peaks centered at 719.3 and 728 eV (Figure 2j), corresponding to the Fe_2_O_3_ characteristic peaks [42].

### 2.2 Electronic Skin

**FIGURE 3:**
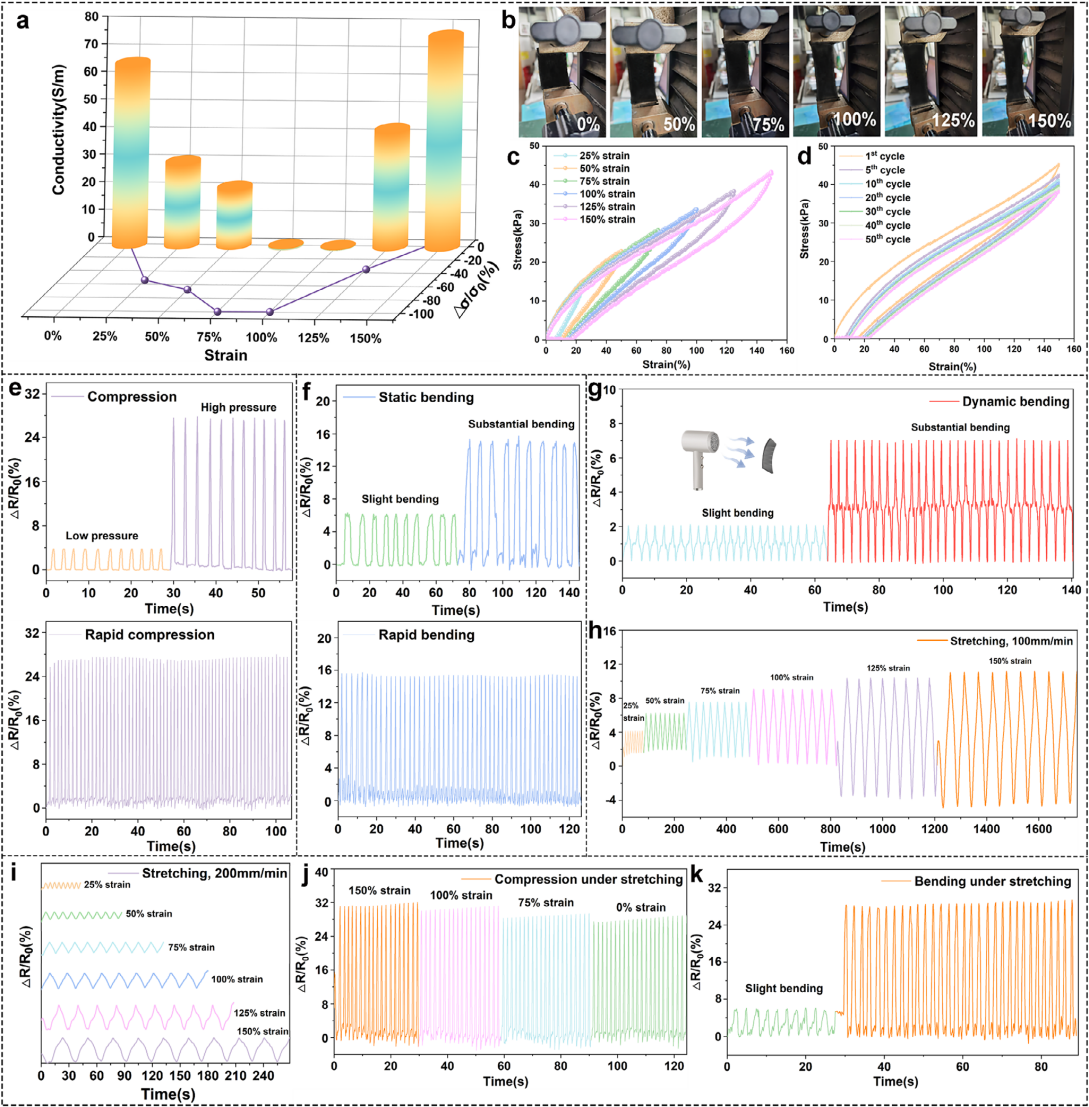
Mechanical and multimodal sensing properties of the RATS‐E‐skin. (a) Electrical conductivity and conductivity percentage of the RATS‐E‐skin as functions of tensile strain. (b) Photographs of the RATS‐E‐skin with increasing tensile strain. (c) Tensile stress–strain curves of the RATS‐E‐skin at 0%–150% strain. (d) Cyclic tension–recovery curves for 50 cycles at a 150% fixed strain. Relative resistance changes of the RATS‐E‐skin under (e) different pressure magnitudes and loading rates, (f,g) static and dynamic bending processes, and (h) cyclic stretching with a consistent tensile rate of 100 mm/min at different strains. (i) Resistance responses of the RATS‐E‐skin under different strains, with a tensile rate of 200 mm/min. Relative resistance changes of the RATS‐E‐skin under (j) compression at different stretching degrees, (k) bending with a fixed stretching condition.

To achieve intelligent electromagnetic protection, the sensing capability of the RATS‐E‐skin was investigated. Because electrical conductivity crucially determines the sensing capability, the electrical conductivity of the E‐skin was evaluated at various tensile strains, as shown in Figure 3a. With the tensile strain increases from 0% to 150%, the electrical conductivity initially declined and subsequently rose, exhibiting a non‐monotonic trend. At a tensile strain of 0%–100%, the LM's oxide layer did not rupture. The scaly and adjacent overlapping graphene sheets slid, the distance between the sheets increased, and the Fe_2_O_3_ nanorods dispersed from clusters to independent small rods, decreasing the electrical conductivity. When the tensile strain exceeded 100% (125%–150%), the surface stress reached the critical threshold that the LM could withstand, the outer oxide layer was damaged, and the spheres were pulled apart to metal wires, connecting the conductive network to a conductor and further enhancing the electrical conductivity [43, 44].

Furthermore, good mechanical properties can ensure that the material operates stably in a complex environment while simultaneously affecting the accuracy and reliability of the sensing. Therefore, cyclic tensile–release tests were conducted on the E‐skin at different strains (Figure 3b). Figure 3c presents the stress–strain curves of the E‐skin at 25%, 50%, 75%, 100%, 125%, and 150% stretching. Clearly, the maximum tensile stresses were 15.3, 22.9, 28.8, 33.8, 38.5, and 43.5 kPa, respectively, and the tensile strength continuously increased with gradually increasing tensile strain. Higher tensile strains generated higher elastic hysteresis, indicating that the energy dissipation mechanism was more effective during stretching [45]. The cyclic tensile test results in Figure 3d show that the E‐skin repeatedly elongated at a strain of 150% possessed a certain shape memory. Although the first tensile cycle showed relatively obvious hysteresis, the molecular chains gradually established a more efficient response mechanism in subsequent cycles, enabling faster adaptation to stress changes, shortening the relaxation time, and reducing hysteresis [46]. After 50 cycles, the stress reduction was negligible, indicating the E‐skin's structural stability and durability.

Drawing inspiration from the adaptive responses of pholidota to diverse environmental stimuli, this study establishes functional analogies for the RATS‐E‐skin when deployed as deformable equipment armor in complex electromagnetic warfare scenarios. The material's pressure sensitivity was evaluated through cyclic loading tests simulating environmental impacts, spanning low‐pressure regime (≈1 N) and high‐pressure condition (≈60 N) (Figure 3e). Resistance changes demonstrated distinct responses, attributed to microstructural reorganization under compression. Reduced interlayer distances between graphene sheets, expanded contact areas, and tighter coupling with Fe_2_O_3_ nanorods, form improved conductive pathways that facilitate electron transport. Concurrently, LM droplets may coalesce under high‐pressure, thereby enhancing the conductive network. Notably, the E‐skin exhibited velocity‐dependent sensitivity during rapid pressure application, mirroring the dynamic force variations in natural impacts. The material's bending sensitivity was evaluated through bending tests simulating pholidota's defense postures. Static bending tests were first conducted, spanning small‐bending angle (≈30°) and large‐bending angle (≈60°) induced measurable resistance changes (Figure 3f). During bending, compression occurs on the inner arc while tension develops on the outer arc [47]. Increased bending angles significantly amplified the resistance variation, which recovered periodically upon straightening. Rapid bending simulations further demonstrated the E‐skin's capability to detect bending velocity. Dynamic bending tests were further performed by applying airflow from a hair dryer to the E‐skin to simulate dynamic bending scenarios. The results in Figure 3g showed that under slight dynamic bending (corresponding to low wind speed), the E‐skin exhibited a relative resistance change rate of approximately 2.18%. Under substantial dynamic bending (corresponding to high wind speed), the relative resistance change rate increased to approximately 7.02%. This ability to sense pressure magnitude, rate, and curvature is vital for equipment protection. For deformable armor, low‐pressure detection enables preemptive hazard avoidance, while high‐pressure/bending monitoring facilitates critical safety interventions such as impact mitigation and structural integrity preservation.

**FIGURE 4:**
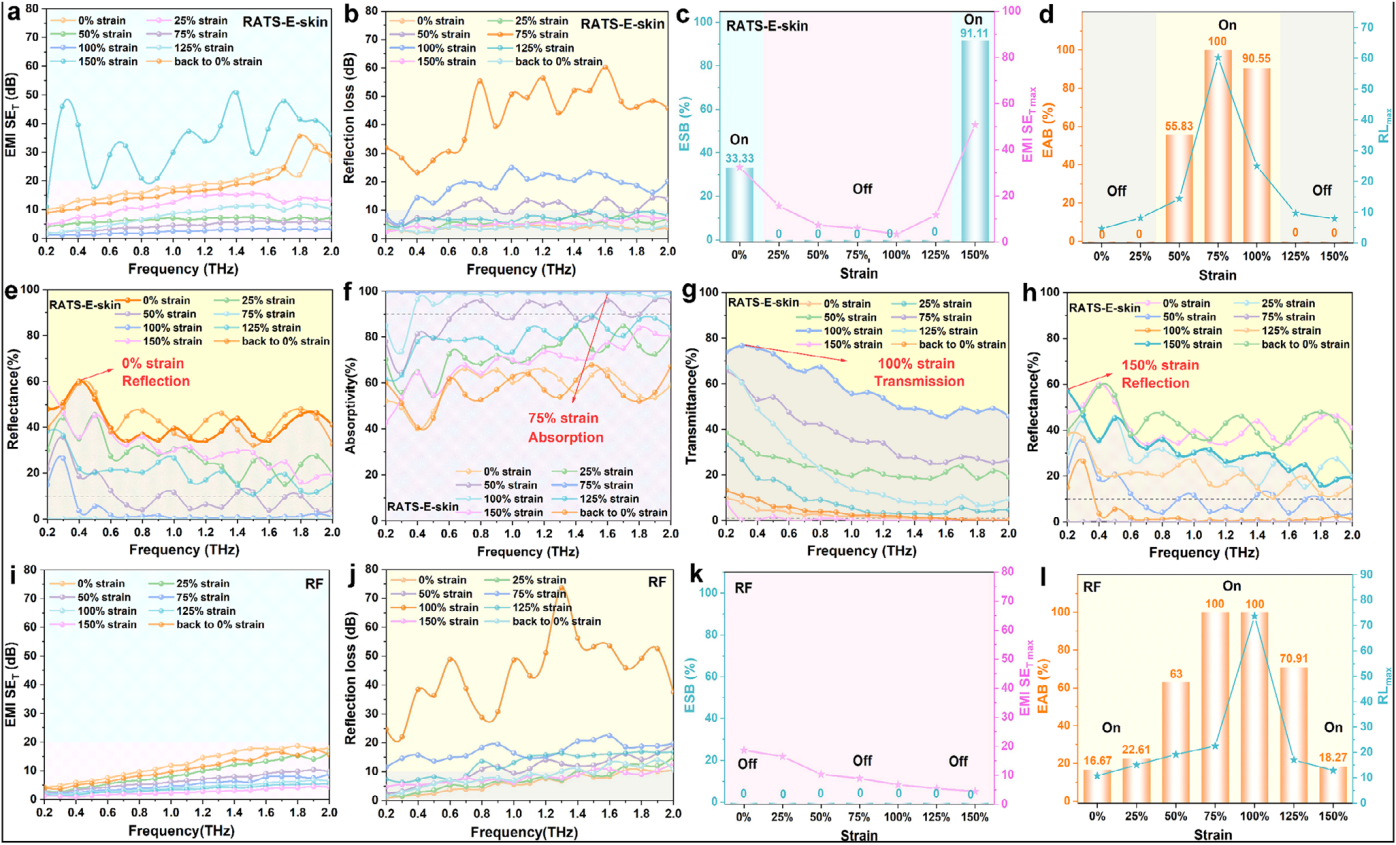
Switchable terahertz properties of the RATS‐E‐skin. (a) EMI SE_T_ and (b) RL curves of the RATS‐E‐skin at different tensile strains. (c) Effective shielding bandwidth (ESB) and EMI SE_Tmax_ and (d) effective absorption bandwidth (EAB) and RL_max_ values of the RATS‐E‐skin under 0%–150% strain, demonstrating the cyclical “on‐off‐on” shielding and “off‐on‐off” stealth switching. (e,h) Reflectance and (f) absorptivity and (g) transmittance curves of the RATS‐E‐skin at different tensile strains, showing four‐state reversible switching of terahertz‐wave reflection, absorption, transmission, and secondary reflection. (i–l) Comparison of the LM‐free RF's absorption and shielding properties.

Pholidota's scale expansion and contraction mechanisms offer a model for armor stretching deformations. Figure 3h and Figure S3 show the responsivity of the RATS‐E‐skin when the applied tensile strain is gradually increased from 25% to 150% (in 25% increments) at a tensile rate of 100 mm min^−1^. These test results are in perfect correspondence with the conductivity variation trend in Figure 3a. At tensile strains from 25% to 100%, the resistance of the stretched E‐skin was higher than that of the unstretched E‐skin, possibly because of the decreased electrical conductivity at this stage; the resistance decreased and recovered to a level close to the initial value after strain release, forming a stable cycle. At tensile strains of 125% and 150%, the resistance first increased and then decreased after the initial stretching, which corresponding to the tendency of electrical conductivity to first decline and subsequently rise. When the tensile strain exceeded 100%, the LM's oxide layer began cracking, and the electrical conductivity recovered gradually, and the resistance decreased accordingly. With continued cyclic stretching and release, the LM's oxide layer fully cracked and entered an activated state. During stretching, the metallic wires elongated to bridge the graphene sheets leading to a decrease in resistance; during release, the metallic wires contracted and some of the bridges broke, causing the resistance to rise, and the resistance change remained stable during the subsequent cyclic stretching and releasing. When the tensile strain was removed, the E‐skin's resistance was slightly higher than that of the initial unstretched E‐skin and was probably slightly affected by the stretching‐generated microcracks, yet the E‐skin maintained a certain level of electrical conductivity at all times. Figure 3i and Figure S4 compare the E‐skin's cyclic sensing behaviors at different tensile rates. At both 100 and 200 mm min^−1^ the resistance responses were stable, indicating rate‐independent sensing. At tensile strains of 25%, 50%, 75%, 100%, 125%, and 150%, the responses were distinguishable and repeatable (Figure S5). After the detector built into the equipment completes the collection of electromagnetic signals, the E‐skin receives the demand and can accurately determine the target strain value required for the electromagnetic function according to the preset strain‐terahertz function matching rules, thereby issuing corresponding instructions. For instance, 0% strain corresponds to a high‐reflection protective state, 75% strain corresponds to a high‐absorption stealth state, and 100% strain corresponds to a high‐transmission communication state (the terahertz performance data of the E‐skin under different strains are shown in Figure 4). To intuitively demonstrate the precise deformation perception capability of the E‐skin serving as the deformable equipment armor, a targeted simulation verification experiment was designed (Figure S6). The E‐skin was tightly attached to the surface of a balloon, which was equivalent to the main body of the deformable equipment. The gradual inflation of the balloon drove the E‐skin to generate tensile deformation. During the 16 s balloon inflation process, the relative resistance change of the E‐skin exhibited a significant monotonous upward trend. This result directly indicates that the E‐skin can accurately perceive the tensile deformation process of the main body of the deformable equipment through the dynamic changes in its own resistance. The design logic of this simulation experiment is fully consistent with the actual working mechanism of the deformable equipment armor, and such perception capability is the prerequisite for realizing electromagnetic function switching. Only by capturing the deformation degree of the equipment in real time can the drive unit be instructed to start or stop timely, ensuring that the equipment is accurately switched to the target function.

However, environmental conditions are complex and variable. Pholidota's exposure to predators during varying scale stretches mirrors deformable equipment under attack during active strain application, armor thus demands full‐process, all‐round perception under complex conditions. The E‐skin's gradual release from 150% to 100%, 75%, and 0% strains was simulated while applying pressure, and the results revealed that although the resistance change gradually decreased, the change magnitude substantially increased compared with that during only stretching (Figure 3j). Next, the E‐skin stretched to a certain strain and subsequently bent was also simulated, and the resistance change's sensitivity was greatly enhanced compared to that when the E‐skin was only bent (Figure 3k). This sensing capability was critical for armor: the stretched E‐skin, upon release to 100%, 75%, and 0% strain, exhibited transmission, absorption, and reflection states respectively (see Figure 4 for detailed data), corresponding to armor's retained perception in equipment's attack, concealment, and protection modes, to enhance offensive and defensive measures under stealth or shielding conditions. Moreover, the armor's capability to detect high deformations (such as strains of 100%–150%) could be used to achieve more flexible functions. For example, armor could real‐time monitor large deformations of equipment under external forces like impacts and collisions, feeding timely feedback to the control system to ensure equipment safety. These sensitive and stable multimodal sensing capabilities of RATS‐E‐skin verified its perception potential as an electronic skin, enabling it to monitor stimulus signals in real time. Among these capabilities, pressure and bending sensing verify its potential as an electronic skin, while tensile strain acts as the variable that drives RAT function switching. After an external electromagnetic signal is input, the E‐skin issues deformation instructions. The microdrive unit built into the equipment receives the instructions and actively applies strain, which in turn drives the E‐skin to deform. During the deformation process, the E‐skin continuously monitors its own deformation degree via its resistive characteristics. Once it verifies that the characteristic resistance variation value corresponding to the target strain is achieved, it sends a feedback instruction to stop the operation of the drive unit, and the terahertz characteristics switch to the corresponding electromagnetic function. In summary, the strain‐dependent terahertz characteristics serve as the core of functional output, and the strain‐dependent resistive characteristics are the key means to realize this function, their synergistic effect jointly enables closed‐loop defense.

### 2.3 “Reflection–Absorption–Transmission” Switchable Terahertz Functions

Pholidota's instinctive scale contraction–expansion in response to external stimuli simulates the process in which the deformable equipment is triggered to actively deform after the equipment detector identifies electromagnetic signals, thereby enabling effective terahertz wave RAT switching and immediate protection. Establishing the correlation between strain values and terahertz electromagnetic properties is the basis for achieving the target functions, and provides performance guidance for the E‐skin to issue deformation instructions.

Figure 4 shows the terahertz characteristics of the RATS‐E‐skin at different tensile strains. In the terahertz band, materials’ absorption and shielding properties can be quantified based on the RL and EMI SE_T_, calculated in the reflection and transmission modes, respectively, as follows [48, 49]

(1) $$\mathrm{RL}\;=-10\;\times \;\mathrm{lg}\left[\lim_{n\to \infty }\frac{{E_{r}}^{2}\left(\omega \right)}{{E_{0}}^{2}\left(\omega \right)}\right]$$

(2) $$\mathrm{EMI}\;SE_{T}=-10\;\times \;\mathrm{lg}\left[\lim_{n\to \infty }\frac{{E_{t}}^{2}\left(\omega \right)}{{E_{0}}^{2}\left(\omega \right)}\right]$$

where *n*, *Er(ω)*, *Et(ω)*, and *E_0_(ω)* represent the number of reflections/transmissions in the sample and the reflected, transmitted, and incident intensities, respectively. When the absorptivity exceeds 90%, that is, RL exceeds 10 dB, the material possesses absorption properties. When the transmittance is below 1%, meaning that EMI SE_T_ exceeds 20 dB, the material possessed shielding properties and can satisfy commercial requirements [50, 51]. The corresponding terahertz band is defined as the material's effective absorption bandwidth (EAB) or effective shielding bandwidth (ESB). The reflectance, *R(ω)*, is equal to *1 − A(ω) − T(ω)*, where *A(ω)* and *T(ω)* represent the absorptivity and transmittance, respectively. In the reflection mode, because an aluminum mirror is placed behind the sample to prevent wave propagation, the transmittance (*T(ω)* = 0) is not considered; that is, *R(ω) = 1 − A(ω)*. Therefore, for effective reflection, RL falling below 10 dB is the criterion [52]. Figure 4a,b clearly shows that for the unstretched E‐skin, the agglomerated LM droplets form discontinuous but locally connected conductive regions, and all the EMI SE_T_ values were above 20 dB in the range 1.4–2.0 THz. At 1.9 THz, the transmittance was minimized (0.05 773%), and EMI SE_T_ reached 32.4 dB, indicating certain shielding. Across the entire 0.2–2.0 THz range, the reflectance was above 30%, and the corresponding RL values were uniformly below 5 dB, indicating the complete lack of absorption and exhibition of the “reflection mode” (Figure 4e). When the E‐skin was stretched, the transmittance and reflectance increased and declined, respectively. When the strain reached 75%, the absorption was full frequency, and the absorptivity exceeded 99%. All the corresponding RL values exceed 20 dB, and the maximum RL reached as high as 60.2 dB, indicating the “absorption mode” (Figure 4f). When the E‐skin was stretched to a strain of 100%, the stretching of the substrate causes the conductive contact points between the agglomerated droplets to break, leading to an increase in electromagnetic wave transmission channels, the transmittance rose and fluctuated in the range 50%–80%, peaking at 76.7%. The corresponding EMI SE_T_ value plummeted to a mere 1.1 dB, and the shielding completely disappeared, transitioning to the “transmission mode” (Figure 4g). When the strain exceeded 100%, the transmittance began decreasing. When the strain reached 150%, the oxide layer of LM droplets ruptures and is stretched into metallic wires, forming new conductive paths between graphene sheets (Figure S7), the E‐skin's electrical conductivity sharply increased, while the transmittance decreased to approximately zero. The maximum EMI SE_T_ values remarkably increased to 50.8 dB, while all the RL values remained below 10 dB, indicating reversion to the “reflection mode” (Figure 4h). When the E‐skin's stretch was released and the strain returned to 0%, the LM contracted because of its fluidity as the matrix rebounded. However, because the surface oxide layer had already been damaged, it still possessed strong electrical conductivity. When the LM was activated for 1 week, its electrical conductivity decreased, presumably because the LM spin‐coated on the matrix surface contacted oxygen and was re‐oxidized, forming an oxide layer [53, 54]. The LM's oxidation was accelerated by ultrasonicating the E‐skin in an ethanol solvent (the measured electrical conductivity substantially decreased after 3 min of ultrasonication). Ultrasonic cavitation, acoustic streaming, and micro‐perturbation enhanced oxygen diffusion from the solution to the LM surface, increased the reaction activity, and enabled the rapid reformation of the oxide layer covering the LM surface, restoring the LM to its initial state. The EDS spectrum in Figure S8 shows the LM's morphology in the E‐skin that was stretched and subsequently unstretched. The main components, Ga and In, were uniformly distributed within the LM spheres, and the oxide layer was distributed around the LM's main body, which main component was Ga_2_O_3_. Evidently, according to Figure 4a,b, the RL and EMI SE_T_ values of the re‐oxidized and unstretched E‐skins almost overlapped, suggesting the utilization of the E‐skin's switching and cycling of the terahertz‐wave “reflection‐absorption‐transmission‐secondary reflection” mode through re‐oxidation. At 0%, 75%, 100%, and 150% tensile strains, the EAB percentages reached 0%, 100%, 90.55%, and 0%, respectively, enabling the “off‐on‐off” conversion with both the conventional path (0%‐100%‐0%) and the extreme path (0%‐100%‐150%) for absorption performance (Figure 4c). Simultaneously, the ESB percentages reached 33.33%, 0%, 0%, and 91.11%, respectively, achieving the “on‐off‐on” conversion with both the conventional path (0%‐100%‐0%) and the extreme path (0%‐100%‐150%) for shielding performance (Figure 4d). In particular, the reactivation of shielding performance under 150% strain adds an emergency electromagnetic protection mechanism for equipment against extreme stress impacts. Tabe 1 lists the properties of some electromagnetic functional materials. Compared with existing materials that only achieve two‐state switching (absorption‐reflection or absorption‐transmission), the RATS‐E‐skin realizes for the first time the four‐state dynamic switching covering the entire 0.2–2.0 THz frequency band (Figure 4e–h) (Table 1).

**TABLE 1:** Comparison of the properties among electromagnetic functional materials in open literature.

Materials	Regulated mechanism and function switching capability	Regulated band	Maximum change for reflection loss (△RL) (dB)	Maximum change for EMI shielding effectiveness (△EMI SE_T_) (dB)	Maximum reflection loss(dB)	Effective absorption bandwidth (RL>10 dB)	Maximum EMI shielding effectiveness (dB)	Effective shielding bandwidth (EMI>20 dB)	Refs.
RATS‐E‐skin	**Strain, four‐state switching (R‐A‐T‐R’)**	**0.2**–**2.0 THz**	**55.52**	**47.39**	**60.24**	**1.8 THz (100%)**	**50.81**	**1.64 THz (91.1%)**	**This work**
PEDOT:PSS/cellulose aerogel	Redox, two‐state switching (A‐T)	0.2–1.2 THz	N/A	16.53	N/A	1 THz (100%)	16.99	0 THz	[15]
Ti_3_C_2_T*_x_* film with wrinkled structures	Strain, two‐state switching (R‐T)	0.2–1.6 THz	N/A	9.1	N/A	N/A	15.2	0 THz	[31]
Ti_3_C_2_T*_x_* MXene sponge foam	Single‐state regulation (A)	0.3–1.65 THz	0	0	65	1.35 THz (100%)	40	1.35 THz (100%)	[26]
Zn^2+^ MXene foam	Single‐state regulation (A)	0.2– 2.0 THz	0	0	15	1.14 THz (63.3%)	51	1.8 THz (100%)	[49]
PNIPAM/PEDOT: PSS/MXene aerogel	Temperature/light, two‐state switching (R‐T)	8.2–12.4 GHz	N/A	43.8	N/A	N/A	59.3	4.2 GHz (100%)	[16]
rGO/VO_2_ aerogel	Temperature, two‐state switching (A‐R)	9.0–16.27 GHz	49	N/A	64	7.27 GHz (45.4%)	N/A	N/A	[17]
Functional carbon spring	Strain, two‐state switching (A‐R)	4.64–18.0 GHz	30	N/A	35	13.36 GHz (83.5%)	N/A	N/A	[18]
Bionic octopus‐inspired C/Fe_3_C foam	Strain, three‐state switching (R‐A‐R)	10.2–18.0 GHz	47.6	N/A	57.6	7.8 GHz (48.75%)	N/A	N/A	[19]
GO/QC hydrogel	Temperature, two‐state switching (A‐T)	12.0–18.0 GHz	43.99	N/A	53.99	6 GHz (37.5%)	N/A	N/A	[20]
PANI/SA hydrogel	Solvent, two‐state switching (A‐T)	8.2–12.4 GHz	N/A	22.8	N/A	N/A	25.8	4.2 GHz (100%)	[21]
PMCP hydrogel	Temperature, two‐state switching (A‐T)	8–12 GHz	N/A	44.6	N/A	N/A	53.9	4 GHz (100%)	[22]
M‐organohydrogel	Temperature, two‐state switching (A‐T)	12.4–18.0 GHz	N/A	38	N/A	N/A	39.3	5.6 GHz (100%)	[23]
Magnetic liquid metal film	Strain, two‐state switching (R‐T)	8.2–12.4 GHz	N/A	60	N/A	N/A	80	4.2 GHz (100%)	[24]
rGO/PPy/PUF	Strain, two‐state switching (A‐T)	8.2–12.4 GHz	N/A	32.5	N/A	N/A	40	4.2 GHz (100%)	[25]

To enhance the shielding capability of the unstretched E‐skin, an attempt was made to increase the systemic LM content. The higher‐LM‐content film (where the LM–rGO ratio was 3:1) was densely distributed in the form of large‐sized agglomerates. This structural feature enabled it to form a denser conductive network in the unstretched state, enhancing the reflection of electromagnetic waves. Its maximum EMI SE_T_ reached 36.2 dB (Figure S9), which was slightly increased compared to that of the unstretched E‐skin with a ratio of 2:1, and the ESB percentage increased from 33.3% to 44.4%. When the film was stretched, the number of contact points between droplets decreased, yet the transmittance initially continued rising. When the strain reached 100%, the transmittance was maximized, but the residual conductive network of high‐areal‐density agglomerates could still block part of the electromagnetic waves. Thus, in contrast to the E‐skin with a ratio of 2:1, which possessed transmittances in the range 50%–80%, the film with a ratio of 3:1 possessed transmittances that declined and fluctuated in the 40%–60% range. Usually, when a material's transmittance exceeds 50%, the material possesses good electromagnetic wave transmittance. In the high‐frequency (1.4–2.0 THz) band, the film prepared using an LM–rGO ratio of 3:1 failed to switch to the “transmission mode.” At 150% strain, a large number of agglomerated droplets were stretched to form bridged conductive paths (Figure S10), further strengthening the connectivity of the conductive network, and EMI SE_T_ rose to 53.2 dB. The LM‐deposition method also affects the electromagnetic properties of the films. After dispersion in an octadecanethiol/ethanol solution, LM is uniformly distributed as tiny droplets (Figure S11), with a shielding effectiveness of only 19.0 dB in the unstretched state and no characteristic of a sharp increase in conductivity after stretching (Figure S12). Direct addition of LM tends to cause agglomeration, which is more conducive to strong electromagnetic wave reflection and the reconstruction of the conductive network after stretching, leading to a better switchable electromagnetic performance. Table S1 comprehensively compares the morphology and shielding performance of the films under different LM contents and deposition conditions, intuitively presenting the regulation law of the preparation process.

To determine the systemic LM's role in increasing the E‐skin's electrical conductivity, the terahertz characteristics of the LM‐free RF were compared at various tensile strains. Figure 4i shows that the RF's EMI SE_T_ values obviously remained below 20 dB throughout the entire tensile test, indicating that the RF lacked shielding. With increasing tensile strain, the transmittance invariably increased. Even when the strain exceeded 100%, the transmittance continued rising, without suddenly decreasing, unlike it did in the LM‐containing E‐skin (Figure S13a). This stark contrast fully revealed the important systemic role of the LM in increasing the E‐skin's electrical conductivity. Although the LM's surface was wrapped by an unstretched oxide layer, the LM still possessed a certain degree of electrical conductivity because of factors, such as LM's high electrical conductivity and the movement of the internal electrons. Coupled with the combined effects of the highly concentrated graphene sheets arranged in an overlapping manner and the closely connected cluster‐like Fe_2_O_3_ nanorods, the initial unstretched E‐skin possessed shielding. During the tensile test, the LM gradually changed from the initial droplets to an elongated metallic wire because of the LM's fluidity. When the tensile strain exceeded 100%, the surface stress that the LM could withstand reached a critical threshold, and the oxide layer ruptured. Consequently, the number of electron conduction paths originally blocked by the oxide layer increased, sharply enhancing the electrical conductivity. As shown in Figure 4j, in the 1.7–2.0 THz range, the unstretched RF's RL exceeded 10 dB. Across the high‐frequency range (1.2–2.0 THz), the unstretched RF possessed relatively low reflectivity (Figure S13b), failing to switch to the “reflection mode.” Because of the separation of the conductive components and the combined effects of both absorption components, namely, the rGO and Fe_2_O_3_ nanorods, the reflectivity of the stretched RF initially declined steadily and approached zero. At a 75% strain, the stretched RF possessed full‐frequency absorption. When the strain reached 100%, the RF's absorption was optimized (Figure S13c). With persistent stretching, the RF's reflectivity started increasing. However, in the high‐frequency range, RL remained above 10 dB. In addition, the increased reflectivity was not caused by the LM‐activation‐induced enhanced electrical conductivity. On the contrary, during the RF tensile test, the electrical conductivity consistently decreased because only the distance between the graphene sheets and Fe_2_O_3_ nanorods changed. When the tensile strain reached 150%, the RF's transmittance clearly reached an extremely high level, indicating that the graphene sheets were completely separated and that numerous terahertz waves had passed through, instead of being fully absorbed by, the material. According to the formula *R(ω) =* 1 *− A(ω)* (*T(ω)* = 0), in the reflection mode, the absorptivity's decline increased the reflectivity. When the stress was released, the RL value was slightly higher than that of the unstretched RF. In contrast, the EMI SE_T_ value was slightly lower than that of the initial unstretched RF, indicating that the RF did not fully revert to its original unstretched state and further emphasizing the LM's crucial role. For the stretched and recovered RF, the lack of an LM filling means that the gaps between the separated graphene sheets cannot be filled, thereby decreasing and increasing the reflectivity and transmittance, respectively. In contrast, for the restored RATS‐E‐skin, the LM rebounded with the matrix and reverted to its original droplet morphology, connecting the gaps between the graphene sheets in series and enabling the cyclic regulation of the switchable electromagnetic properties. To test this hypothesis, a pure LM film was also prepared, and its terahertz characteristics were evaluated at different tensile strains (Figure S14). Throughout the entire tensile test, the pure LM film possessed shielding (EMI SE_T_ > 20 dB, RL < 10 dB), which was ascribed to its excellent electrical conductivity. Similarly, for the stretched pure LM film, the transmittance initially ascended and subsequently descended, while the reflectivity first dropped and then rose, suggesting that the obtained results were not accidental. In summary, the LM‐free RF not only failed to switch the shielding from on to off and on again, with the shielding remained in the “off mode” (Figure 4k), but also could not switch the stealth from off to on and off again, with the stealth remained in the “on mode” (Figure 4l). To clarify the function of Fe_2_O_3_ nanorods in the ternary composite, the terahertz properties of the rGO/LM film without Fe_2_O_3_ nanorods under different tensile strains were compared (Figure S15). At 0% strain, the binary composite without Fe_2_O_3_ nanorods fluctuation around 30% (10% lower than the ternary composite with Fe_2_O_3_), which proved that Fe_2_O_3_ nanorods enhance reflection. Figure S16 shows numerous Fe_2_O_3_ nanorods attached between the graphene sheets are tightly connected in the unstretched state, leading to an increase in contact points. These elongated rod‐like Fe_2_O_3_ nanorods with a certain aspect ratio construct a large number of electromagnetic wave scattering units in the graphene nanosheets, form abundant heterointerfaces with the graphene nanosheets, and induce multiple directional scattering of electromagnetic waves at the interfaces, which effectively enhances the interfacial reflection. At 75% strain, the maximum RL value of rGO/LM only reached 32.3 dB (27.9 dB significantly lower than the ternary composite with Fe_2_O_3_ nanorods), this is attributed to the fact that the rod‐like morphology of Fe_2_O_3_ nanorods can construct abundant rGO‐Fe_2_O_3_ and LM‐Fe_2_O_3_ heterointerfaces with rGO sheet and LM spherical in different directions, generating a strong interfacial polarization effect. At 100% strain, as the spacing between graphene sheets increased significantly, the film thickness became thinner, making it easier for electromagnetic waves to penetrate the material. Owing to their tiny size and uniform dispersion between the layers, the Fe_2_O_3_ nanorods exerted a negligible effect on the maximum transmittance with a slight change of ∼5% even though they are also dispersed into independent small rods (Figure 1e). When stretched to 150% strain, the rGO/LM without Fe_2_O_3_ nanorods only achieved 38.9 dB (11.9 dB lower than the ternary composite with Fe_2_O_3_ nanorods). This is because Fe_2_O_3_ nanorods form abundant heterogeneous interfaces with graphene sheets and LM, which further improve electromagnetic shielding performance through multi‐heterogeneous interface polarization and multiple scattering.

### 2.4 Effect of the Number of Layers on the Switchable Terahertz Properties

**FIGURE 5:**
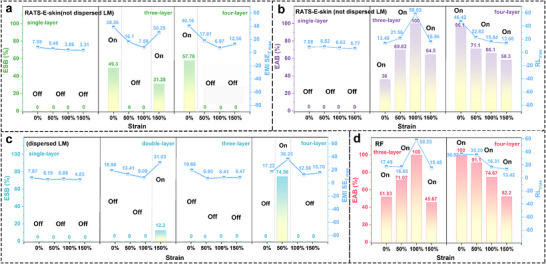
Comparison of terahertz‐wave switchability for composite films containing different numbers of layers. (a) ESB and EMI SE_Tmax_ and (b) EAB and RL_max_ values of the single‐, three‐, and four‐layer rGO/Fe_2_O_3_/LM films (containing directly mixed LM) under different tensile strains. (c) ESB and EMI SE_Tmax_ values of the single‐, double‐, three‐, and four‐layer rGO/Fe_2_O_3_/LM films (containing dispersed LM) under different tensile strains. (d) EAB and RL_max_ values of the LM‐free three‐ and four‐layer rGO/Fe_2_O_3_ films under different tensile strains.

The terahertz‐wave switchability of the films containing different numbers of layers were further investigated. The E‐skin that switched the electromagnetic properties was obtained by spin‐coating two layers. The first layer of the rGO/Fe_2_O_3_/LM coating was uniaxially stretched to a strain of 50% and then fixed. Then, the second layer of the coating was spin‐coated, and the film was further stretched to a strain of 100%. Then, the tension was released, producing an overlapping scaly structure. Figure 5a,b shows the terahertz characteristics of the films prepared by spin‐coating one, three, and four layers. The unstretched single‐layer film already possessed relatively high transmittance and reflectivity (Figure S17). When the films were stretched, both the transmittance and reflectivity continuously rose, and both the shielding and stealth remained in the “off mode,” likely because the content of each shielding or absorptive component in the single‐layer film was too low to block any electromagnetic waves, allowing most of the terahertz waves to penetrate the material. For the unstretched three‐layer film, EMI SE_T_ exceeded 20 dB in the range 1.26–2.0 THz, indicating that the film possessed a certain shielding capability. When the three‐layer film was stretched, the transmittance also initially rose (although only negligibly) and subsequently declined (Figure S18). The initial unstretched three‐layer film's reflectivity was lower than that of the initial unstretched double‐layer film. While the three‐layer film was stretched, the switching between reflection and absorption was also observed. When the tensile strain reached 100%, the three‐layer film possessed full‐frequency absorption. However, when the strain reached 150%, RL exceeded 10 dB in the range 0.84–2.0 THz, and the film's absorption failed to switch back to total reflection. The three‐layer film's absorption persistently remained in the “on mode.” The unstretched four‐layer film also possessed shielding. In the 0.96–2.0 THz range, EMI SE_T_ was above 20 dB. The reflectivity is extremely low, and RL remained above 10 dB across the entire frequency range, indicating that the shielding of the initial unstretched four‐layer film was dominated by absorption rather than high electrical conductivity. However, although the transmittance of the stretched four‐layer film initially increased and then decreased, the increase range was even smaller, and the maximum transmittance was lower, than those of the stretched three‐layer film, fluctuating between 20% and 40% (Figure S19). Additionally, the four‐layer film's mode did not switch between reflection and absorption. The stretched four‐layer film's reflectivity increased, and the film consistently maintained a certain absorption capability, remaining in the “on mode.” Furthermore, as Figures S17–S19 show, because of the LM, the single‐, three‐, and four‐layer films returned to their initial unstretched states when the stress was released, further suggesting that these films’ modes could be repeatedly switched. In summary, the number of spin‐coating layers substantially impacted the terahertz characteristics because the gradually decreased initial reflectance of the three‐ and four‐layer films (compared to that of the double‐layer film) may be because the increased number of spin‐coating layers simultaneously increased the absorbing agent content and film thickness, more likely endowing the initial film with absorption. For the three‐ and four‐layer films stretched to a strain of 75%, the gradually decreased maximum transmittance (compared with that of the double‐layer film) may be because the increased number of layers increased the LM content, enhancing the electrical conductivity. Although the transmittances decreased when the three‐ and four‐layer films were stretched beyond a strain of 100%, when these films were stretched to a strain of 150%, their shielding capabilities were much weaker than that of the double‐layer film, possibly because at a strain of 150%, the LM in the double‐layer film is more likely to form a continuous and uniform conductive pathway, enabling smoother electron transfer and improved electrical conductivity. However, the structures of the three‐ and four‐layer films possessed more defects, such as interlayer separation, because of the increased number of layers. When these films were stretched, more discontinuous regions may have formed, hindering electron conduction and, thus, reducing electrical conductivity.

These were only the terahertz characteristics of the films containing different numbers of layers and prepared using directly mixed LM. In addition, the terahertz‐wave switching of the films containing different numbers of layers and prepared using dispersed LM (Figure 5c and Figure S20) and LM‐free films (Figure 5d and Figure S21) were compared. The initial single‐layer film prepared using dispersed LM also possessed a relatively high transmittance, which increased when the film was stretched. The initial three‐layer film possessed a relatively low transmittance yet marginally higher than that of the three‐layer film containing directly added LM. When the film was stretched, the transmittance first increased and then negligibly decreased, for a reason similar to that describing the double‐layer film's transmittance behavior. Compared to the dispersed LM, the agglomerated LM was far more efficient in shielding the material against the external electric field, and a substantial number of additional effective conductive channels was generated more easily when the film was extensively stretched. For the films containing different numbers of layers and prepared using the pure LM or the directly mixed or dispersed LM, the transmittances suddenly dropped when the films were stretched to a certain strain, indicating that this trend was not random but was determined by the LM's inherent stretching. When the four‐layer film was stretched, the transmittance directly dropped, likely because the number of layers increased and the film's structure was more complex, rendering the film more prone to stress concentration. Additionally, because of its relatively dispersed LM distribution and relatively thin oxide layer, the four‐layer film was easily damaged at the beginning of the stretching. Although the LM‐free three‐layer film clearly switched between the reflection and absorption modes, the reflectivity was quite low for both the unstretched film and the film stretched to a strain of 150%, and RL exceeded 10 dB in the high‐frequency range. The LM‐free four‐layer film did not possess “reflection‐absorption” switching. The reflectivity of the unstretched film was extremely low and increased when the film was stretched. Additionally, the absorption constantly remained in the “on mode.” These results suggest that for the scaly films prepared using two spin‐coated layers, both the component content and number of layers were optimized, and the double‐layer films possessed superior terahertz‐wave regulation. If a single pholidota scale is considered as a unit structure, the double‐layer film's structure is analogous to the coordination between every two adjacent pholidota scales, which not only supplements the component content but also avoids the negative impacts caused by the stacking of excessive unit structures, thereby producing a relatively ideal film for terahertz‐wave switching. The single‐layer film is analogous to pholidota relying on only one scale to function. Because of the simplicity of the unit structure and the limited component content, the single‐layer film may be insufficient for terahertz‐wave switching. The three‐ and four‐layer films are comparable to combining multiple unit structures in a closely stacked manner analogous to that in which multiple pholidota scales closely bond to form a whole. Because pholidota scales cannot move relatively freely, undermining their original natural functions and flexibility, by analogy, issues such as interlayer defects may affect the three‐ and four‐layer films’ terahertz‐wave‐switching capabilities.

### 2.5 The Terahertz‐Wave Switching Mechanism

**FIGURE 6:**
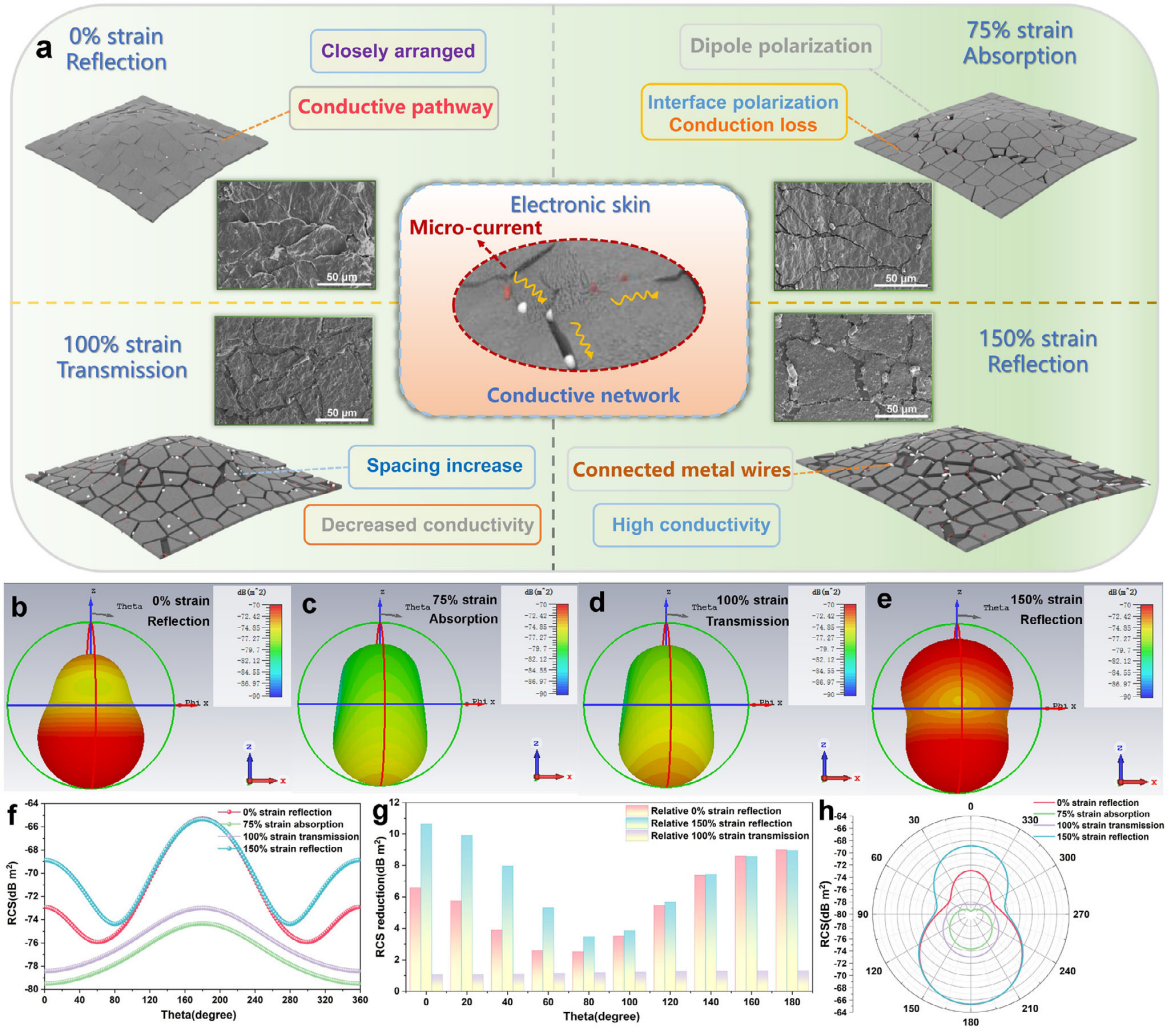
Mechanism and simulation of the terahertz‐wave “reflection‐absorption‐transmission‐secondary reflection” functional switching of the RATS‐E‐skin possessing a bionic pholidota‐inspired structure. (a) Mechanism diagram for the closed‐loop electromagnetic adaptation of RATS‐E‐skin and the corresponding SEM images. (b–e) 3D terahertz‐wave scattering signals of the RATS‐E‐skin during reflection, absorption, transmission, and secondary reflection. (f) RCS curves recorded at different plane‐wave incident angles. (g) RCS reduction in the stealth state relative to the reflection, secondary reflection, and transmission states at different plane‐wave incident angles. (h) RCS radiation patterns of the RATS‐E‐skin during reflection, absorption, transmission, and secondary reflection.

In summary, the bionic pholidota‐structured RATS‐E‐skin combined the functions of an electronic skin with switchable terahertz‐wave RAT functions, integrating perception, defense, stealth, and communication functions (Figure 6a). For the unstretched E‐skin, reflection was derived from the synergistic effect of three factors: the overlapping structure of high‐concentration graphene sheets, the increased contact points brought by tightly connected Fe_2_O_3_ nanorods embedded between the sheets, and the conductive paths formed by the LM connecting them. When the E‐skin was stretched to a strain of 75%, absorption was derived from a combination of multiple electromagnetic wave loss mechanisms, such as interfacial polarization generated by the different interfaces constructed by the rGO sheet, Fe_2_O_3_ nanorod, and LM spherical structures possessing different morphologies and scales, in addition to the multiple reflections and scattering because of the gaps among the graphene sheets during stretching. Specifically, rGO facilitated electron migration/jumping and the formation of conductive networks, while the high conductivity of LM endowed the E‐skin with moderate electrical conductivity, both contributed to conduction loss. Additionally, rGO's oxygen‐containing groups and lattice defects served as polarization centers, triggering multiple dipole polarizations. Structurally, because of their elongated shape, rod‐like structures can interface with sheet‐like and spherical structures in different directions. In addition to the gaps generated during the stretching of the graphene sheets, enabling electromagnetic waves to shuttle back and forth, scattering occurred when the electromagnetic waves encountered the rod‐like structures. The Fe_2_O_3_ nanorods also enhanced the electromagnetic wave scattering, jointly enhancing the weakening of the electromagnetic waves. When the E‐skin was stretched to a strain of 100%, transmission was derived from the substantially wider spaces between the graphene sheets, which hindered the LM filling. Meanwhile, the Fe_2_O_3_ nanorods were dispersed to independent small rods, and the E‐skin thinned with increasing stretching strain, thereby dropping the electrical conductivity. This, in turn, weakened the oscillation of free electrons influenced by the electric field, reducing the reflection. Moreover, as the electron movement generated less heat, the absorption diminished. Consequently, more electromagnetic waves penetrated the material instead of being absorbed and converted to heat energy. Finally, when the E‐skin was stretched to a strain of 150%, reflection was derived from the LM's oxide layer rupturing under tension, forming metal wires, connecting the graphene sheets and Fe_2_O_3_ nanorods in series, and enabling the reconstruction of the electron transmission path, which sharply increased the electrical conductivity. The E‐skin's sensing function was attributed to the conductive network structure comprising rGO, Fe_2_O_3_ nanorods, and the LM. When the E‐skin was compressed or stretched, the distance between the graphene sheets and Fe_2_O_3_ nanorods changed, and the LM's distribution and morphology changed accordingly. Additional conductive contact states constantly formed, detectably changing the resistance and, thus, generating corresponding electrical signals. During this process, the E‐skin always maintained a certain degree of electrical conductivity—only the electrical conductivity's strength changed—and still satisfied the basic conductive mechanism on which sensing depends. Therefore, the E‐skin simultaneously possessed sensing and switchable RAT functions.

To further clarify the RATS‐E‐skin's terahertz‐wave‐switching mechanism, 0.4 THz was selected as the fixed monitoring frequency for calculating radar cross‐section (RCS) simulations. Figure 6b–e shows 3D terahertz‐wave‐scattering signals for reflection, absorption, transmission, and secondary reflection for the RATS‐E‐skin stretched at strains of 0 (unstretched), 75%, 100%, and 150%, respectively. The findings indicate that when the E‐skin was unstretched or maximally stretched, the RATS‐E‐skin's high reflectivity returned terahertz waves along their original paths or scattered them in various directions. This characteristic renders the RATS‐E‐skin more detectable by radar, thereby producing high RCS values. When the strain reached 75%, the RATS‐E‐skin functioned as an effective stealth material, substantially reducing surface reflections under terahertz‐wave radiation and simultaneously substantially weakening the RATS‐E‐skin's terahertz‐wave‐scattering capabilities, thereby notably weakening the signal reflected back to the radar. This behavior was mainly ascribed to the optimized impedance matching, which was further demonstrated by the enhanced and more uniformly distributed absorption electric field (Figure S22). When the E‐skin was stretched to a 100% strain, a large portion of the terahertz waves penetrated the material, producing relatively low‐energy scattering. Nevertheless, compared to the absorption‐dominated scenario, this scenario revealed that under these conditions, the reduced RCS was still less effective. The RCS curves recorded at various plane‐wave incident angles (Figure 6f) reveal that when the RATS‐E‐skin functioned as a stealth material stretched at a 75% strain, it effectively protected against terahertz waves propagating in all directions. The RCS reduction spanned the entire angular range from 0° to 180°. Compared to secondary reflection when the E‐skin was stretched at a 150% strain, the maximum RCS reduction reached 10.63 dB m^2^ (Figure 6g). Similarly, the RCS radiation patterns in Figure 6h offer a vivid visual comparison, highlighting the substantially different terahertz‐wave‐scattering capabilities of the RATS‐E‐skin in its stealth and other modes and strongly indicating the effectiveness of stealth technology in reducing target detectability.

## 3 Conclusion

This work introduces a biomimetic electronic skin (RATS‐E‐skin) featuring an overlapping pholidota‐inspired architecture that achieves dynamic transitions between electromagnetic states across 0.2–2.0 THz at 0%, 75%, 100%, and 150% tensile strains. Overlapping conductive networks enable initial shielding (EMI SE_T_ 32.4 dB), component separation optimizes dielectric loss for broadband absorption (RL 60.2 dB), macroscopic thinning and conductivity suppression facilitate high transmittance (76.7%), and LM oxide‐layer rupture regenerates conductive pathways for secondary shielding (EMI SE_T_ 50.8 dB). Notably, LM's reversible fluidic deformation enables conductivity regeneration at high strain, driving the cyclical “on to off to on” shielding and “off to on to off” stealth switching—with △RL and △EMI SE_T_ of 55.52 dB and 47.39 dB for simultaneous broad‐range adjustment. The synergistic fusion of strain‐resistive sensing with terahertz‐mode regulation establishes an instruction‐execution‐verification active closed‐loop adaptive system, which can autonomously trigger the optimal electromagnetic response, with one typical case being the active switch to a high‐reflection protective state under impact threats. This work presents a versatile material platform for intelligent deformable armor, adaptive stealth surfaces, and wearable systems requiring real‐time stealth‐transmission‐shielding transitions.

## 4 Experimental Section

### 4.1 Materials

Ascorbic acid (C_6_H_8_O_6_), ammonia, absolute ethanol and deionized water (DI) were all provided from Sinopharm Chemical Reagent Co., Ltd. 1‐Octanethiol was purchased from Shanghai Titan Scientific Co., Ltd. The VHB substrate was purchased from 3 M Company. Graphite oxide (GO, with a solid content of 43.42%) was provided by Changzhou Six Element Materials Technology Co., Ltd. Iron oxide nanorod powder (with a width of 20–50 nm and a length of 100–200 nm) was purchased from Qinghe Ruijiang Metal Material Co., Ltd. EGaIn (LM, composed of 75.5% gallium and 24.5% indium) was provided by Huatai Metal Material Technology Co., Ltd.

### 4.2 Fabrication of the rGO/Fe_2_O_3_/LM Hybrid Solution

First, 0.4606 g of graphite oxide was weighed and dispersed in 5 mL of deionized water, and then ammonia was added drop by drop to adjust the pH of the solution to 10.0. Under stirring conditions, 5 mL of the above‐mentioned uniform GO dispersion (40 mg mL^−1^) was thoroughly mixed with 2 g of the reducing agent ascorbic acid. Subsequently, the mixed solution was placed in an ice bath and subjected to ultrasonic treatment using a KQ‐400KDE ultrasonic cleaner at a power of 320 W (80% of the rated power) for 2 h. After that, the obtained mixed solution was transferred into a three‐necked flask, placed in a constant‐temperature oil bath at 80°C, and refluxed for 4 h to complete the reduction process. After the reaction was completed, the resulting black solution was separated by filtration and then washed with deionized water to obtain a uniformly dispersed black rGO solution. 0.16 g of Fe_2_O_3_ powder was added to the rGO solution, followed by ultrasonic treatment lasting for 1 h to obtain the rGO/Fe_2_O_3_ hybrid solution. A certain amount of LM was added to it to prepare rGO/Fe_2_O_3_/LM hybrid solutions with different mass ratios (the mass ratios of LM to rGO were 2:1 and 3:1, respectively). For comparison, a rGO/Fe_2_O_3_/LM hybrid solution obtained after dispersing LM was also prepared. 0.00 143 g of 1‐octanethiol was dissolved in 5 mL of absolute ethanol, a certain amount of LM was added and ultrasonic dispersion was carried out for 2 h to obtain a LM dispersion (1 mmol L^−1^), and then it was mixed with the rGO/Fe_2_O_3_ hybrid solution.

### 4.3 Fabrication of RATS‐E‐skin and Composite Films with Different Layers

First, the plasma was set to evacuate the vacuum for 6 min, and then the elastic substrate was treated for 5 min to make it hydrophilic. 2 mL of the rGO/Fe_2_O_3_/LM hybrid solution was spin‐coated on the treated elastomer at 3000 rpm for 30 s, and then baked in an oven at 130°C for 10 min. The coating was pre‐stretched to a 50% strain and fixed with a fixture. Then, another 2 mL of the rGO/Fe_2_O_3_/LM hybrid solution was spin‐coated at 3000 rpm for 30 s for the formation of the second coating layer, which was baked at 80°C for 1 h and further pre‐stretched to a 100% strain. Finally, the pre‐stretching was released to obtain a double‐layer RATS‐E‐skin with an overlapping scale structure. Three‐, and four‐layer rGO/Fe_2_O_3_/LM films were prepared by a similar method. After pre‐stretching to a 100% strain, the three‐layer was spin‐coated, further stretched to a 150% strain, and then the pre‐stretching was released to obtain a three‐layer film. After pre‐stretching to a 150% strain, the four‐layer was spin‐coated, further stretched to a 200% strain, and then the pre‐stretching was released to obtain a four‐layer film. By changing the rGO/Fe_2_O_3_/LM hybrid solution to a rGO/Fe_2_O_3_ hybrid solution, a pure LM dispersion, and a rGO/LM hybrid solution while keeping other conditions unchanged, a LM‐free rGO/Fe_2_O_3_ film, a pure LM film, and a rGO/LM film were prepared.

### 4.4 Measurement of Terahertz Absorption and Shielding Properties

The reflection and transmission characteristics of the samples were measured using a terahertz time‐domain spectroscopy system (TAS7400TS, ADVANTEST). During the measurement process, a femtosecond fiber laser with a central wavelength of 1550 nm, a pulse width of less than 50 fs, and an output power of greater than 20 mW was selected as the excitation source. With the aid of two femtosecond laser‐pumped optical antennas, the terahertz signals were received through an asynchronous sampling method. The effective frequency range of this system was between 0.2 and 2.0 THz, the spectral resolution was 1.9 GHz, and the test time step was approximately 131 ps. The terahertz light source penetrated the sample at an incident angle of 11°. Before the test was carried out, water removal treatment was performed using nitrogen gas. The test environment was required to be at room temperature of 23°C ± 5°C, and the air humidity was maintained below 5%. The sample was placed on a hollow hole of an aluminum plate, and the diameter of the hollow hole was approximately 1.2 cm. In the reflection mode, an aluminum mirror was placed on the sample to block the propagation of the waves (T = 0). However, in the transmission mode, no aluminum mirror was used. The incident terahertz wave converged at the central position of the sample.

### 4.5 Characterization

The surface morphology of the samples was analyzed by SEM (Quanta 200 FEG). Raman spectra were recorded on a LabRAM HR Evolution Raman spectrometer excited by a 532 nm laser. The crystal structure was characterized by XRD (Rigaku SmartLab SE) equipped with a Cu Kα ray, and the test was carried out at a scanning speed of 2°/min within the diffraction angle range of 10°–80°. The elemental composition and valence states of the elements in the samples were analyzed by XPS (ESCALAB 250Xi). The electrical conductivity of the samples was measured by an M‐3 type handheld digital four‐probe tester (Suzhou Jingge Electronics Co., Ltd.). The sample substrate was treated with a PLASMA CLEANER PDC‐32G, and then the spin‐coating operation was carried out by a KW‐4T desktop spin coater. Tensile tests were carried out on samples with a width of 37 mm and a thickness of 0.52 mm by an electronic universal testing machine (UTM2502), and the tensile speed was set at 100 mm/min to evaluate the mechanical properties of the samples. The sensing properties of the samples were obtained through the coordinated work of an electronic universal testing machine and an electrochemical workstation (CHI660E). The sample was placed in the middle of the fixture of the electronic universal testing machine. A conductive copper sheet and a conductive copper wire were, respectively, connected to both the upper and lower ends of the sample, and at the same time, it was separated by an insulating plastic sheet between the copper sheet and the fixture. The sample was connected to the electrochemical workstation through the conductive copper wire. The electronic universal testing machine was used to carry out stretching and compressing operations on the sample, and the change in resistance was recorded with the help of the electrochemical workstation under a constant voltage of 0.5 V. A square model with a side length of 20 mm was constructed. RCS of the model in four states of reflection, absorption, transmission, and secondary reflection was simulated using CST Studio Suite 2018, with the monitoring frequency set at 0.4 THz. The plane wave was incident along the negative *z*‐axis direction, and the model sample was placed on the XOY plane.

## Conflicts of Interest

The authors declare no conflicts of interest.

## Supporting information




**Supporting File**: advs74646‐sup‐0001‐SuppMat.docx

## Data Availability

Research data are not shared.
