# Supplementary material for: Pholidota‐Inspired Electronic Skin Possessing Terahertz‐Wave Reflection–Absorption–Transmission Switchability
Source: Adv Sci (Weinh). 2026 Jun 3;13(37):e18194. doi: 10.1002/advs.202518194 (PMC13325925; doi:10.1002/advs.202518194)
Supplement: Supplementary file 1 — Supporting File: advs74646‐sup‐0001‐SuppMat.docx [file ADVS-13-e18194-s001.docx]

Supplementary Information for

**Pholidota-Inspired Electronic Skin Possessing Terahertz-Wave Reflection–Absorption–Transmission Switchability**

*Shangjing Li^a^, Jiangsong Hou^a^, Yifeng Ruan^a^, Jiang Du^a^*, and Jun Qiu^a,b^**

S. J. Li, J. S. Hou, Y. F. Ruan, Prof. J. Du

School of Materials Science and Engineering, Tongji University, Shanghai 201804, PR China

dujiang@tongji.edu.cn

Prof. J. Qiu

School of Materials Science and Engineering and Key Laboratory of Advanced Civil Engineering Materials of Education of Ministry, Shanghai 201804, PR China

qiujun@tongji.edu.cn

This PDF file includes:

Experimental calculation (Page 1–3)

Detailed Analysis of Experimental Details (Page 4–5)

Supplementary Figures S1 to S23 (Pages 6–18)

Supplementary Tables S1 to S2(Pages 19–20)

References (Pages 21–23)

**Experimental calculation**

Calculations of Terahertz Absorption System:

The ratio of the whole reflected and transmitted power to incoming power can be written as: ^[1]^

$\lim_{n\to\infty} \frac{{E_{r}}^{2}\left( \omega\right)}{{E_{0}}^{2}\left( \omega\right)}=\frac{\left[ n\left( \omega\right)-1 \right]^{2}+\kappa^{2}\left( \omega\right)}{\left[ n\left( \omega\right)+1 \right]^{2}+\kappa^{2}\left( \omega\right)}+16\times\frac{n^{2}\left( \omega\right)+\kappa^{2}\left( \omega\right)}{\left\{ \left[ n\left( \omega\right)+1 \right]^{2}+\kappa^{2}\left( \omega\right) \right\}^{2}}$ (1)

$\lim_{n\to\infty} \frac{{E_{t}}^{2}\left( \omega\right)}{{E_{0}}^{2}\left( \omega\right)}=16\times\frac{n^{2}\left( \omega\right)+\kappa^{2}\left( \omega\right)}{\left\{ \left[ n\left( \omega\right)+1 \right]^{2}+\kappa^{2}\left( \omega\right) \right\}^{2}}\times\frac{\left\{ \left[ n\left( \omega\right)+1 \right]^{2}+\kappa^{2}\left( \omega\right) \right\}^{2}\times\exp\left( \frac{-2\times\omega\times\kappa\left( \omega\right)\times d}{c} \right)}{\left[ n\left( \omega\right)+1 \right]^{2}+\kappa^{2}\left( \omega\right)-\left\{ \left[ n\left( \omega\right)-1 \right]^{2}+\kappa^{2}\left( \omega\right) \right\}\times\exp\left( \frac{-4\times\omega\times\kappa\left( \omega\right)\times d}{c} \right)}$ (2)

The THz reflectance(*R(ω)*) and transmittance(*T(ω)*) are obtained through fast Fourier transform (FFT) calculation by following equations:

$R\left( \omega\right)={|S_{11}|}^{2}=\frac{{E_{r}}^{2}(\omega)}{{E_{ref1}}^{2}(\omega)}$ (3)

$T\left( \omega\right)={|S_{21}|}^{2}=\frac{{E_{t}}^{2}(\omega)}{{E_{ref2}}^{2}(\omega)}$ (4)

where *E_r_(ω)*, *E_t_(ω)*, *E_ref1_ (ω)*, and *E_ref2_ (ω)* refer to the electromagnetic field intensity of reflection, transmission, and the corresponding references of the sample.

The electromagnetic field intensity of reflection (*E_r_(ω)*) and transmission (*E_t_(ω)*) can be represented as:

${E_{r}}^{2}\left( \omega\right)={E_{r1}}^{2}\left( \omega\right)+{E_{r2}}^{2}\left( \omega\right)+{E_{r3}}^{2}\left( \omega\right)+\ldots+{E_{\mathrm{rn}}}^{2}\left( \omega\right)={E_{0}}^{2}\left( \omega\right)\times{R_{12}}^{2}+{E_{0}}^{2}\left( \omega\right)\times{T_{12}}^{2}\times{T_{21}}^{2}\times{P_{ñ}}^{4}\left( \omega,L \right)+{E_{0}}^{2}\left( \omega\right)\times{T_{12}}^{2}\times{T_{21}}^{2}\times{R_{21}}^{2}\times{P_{ñ}}^{8}\left( \omega,L \right)+\ldots{E_{0}}^{2}\left( \omega\right)\times{T_{12}}^{2}\times{T_{21}}^{2}\times{R_{21}}^{2n-4}\times{P_{ñ}}^{4n-4}\left( \omega,L \right)$ (5)

${E_{t}}^{2}\left( \omega\right)={E_{t1}}^{2}\left( \omega\right)+{E_{t2}}^{2}\left( \omega\right)+{E_{t3}}^{2}\left( \omega\right)+\ldots+{E_{\mathrm{tn}}}^{2}\left( \omega\right)={E_{0}}^{2}\left( \omega\right)\times{T_{12}}^{2}\times{T_{21}}^{2}\times{P_{ñ}}^{2}\left( \omega,L \right)+{E_{0}}^{2}\left( \omega\right)\times{T_{12}}^{2}\times{T_{21}}^{2}\times{R_{21}}^{4}\times{P_{ñ}}^{6}\left( \omega,L \right)+{E_{0}}^{2}\left( \omega\right)\times{T_{12}}^{2}\times{T_{21}}^{2}\times{R_{21}}^{8}\times{P_{ñ}}^{10}\left( \omega,L \right)+\ldots{E_{0}}^{2}\left( \omega\right)\times{T_{12}}^{2}\times{T_{21}}^{2}\times{R_{21}}^{4n-4}\times{P_{ñ}}^{4n-2}\left( \omega,L \right)$(6)

where *E_0_* responds to the initial intensity of terahertz signal, *E_r1_*, *E_r2_*, *E_r3_*, …*E_rn_*, and *E_t1_*, *E_t2_*, *E_t3_*, …*E_tn_* represent the electromagnetic field intensity from one, two, three to n times reflected, and transmitted terahertz signal from the sample to air, respectively.

The propagation factor$P_{ñ}(\omega,L)$ of terahertz signal in the sample is shown as below, which is equal to the ratio of *E_L_(ω)* to *E_0_(ω)*:

$E_{L}\left( \omega\right)=E_{0}\left( \omega\right)\times{P_{ñ}(\omega,L)}^{-iñ\left( \omega\right)\omega L/c}$ (7)

where *E_L_(ω)* is the electromagnetic field intensity of the terahertz wave signal when the terahertz wave propagates in the sample for a length of *L* and attenuates, *c* is the light velocity, *ω* is the angular frequency, and $ñ\left( \omega\right)$ is the complex refractive index, which is generally considered that the complex refractive index of air is approximately equal to 1, that is

$ñ\left( \omega\right)=n\left( \omega\right)-i\kappa$ (8)

where *n(ω)* is the extraction of the real refractive index, and *κ* is the extinction coefficient, which can be calculated by following equations:

$n\left( \omega\right)=\frac{\varphi\left( \omega\right)c}{\omega d}+1$ (9)

$\kappa\left( \omega\right)=\frac{-ln\{T\left( \omega\right)\frac{\left[ n\left( \omega\right)+1 \right]^{2}}{4n\omega}\}}{\omega d}$ (10)

$\varphi\left( \omega\right)={\varphi_{\mathrm{sam}}\left( \omega\right)-\varphi}_{\mathrm{ref}}(\omega)$ (11)

where *φ(ω)*, *φ_sam_ (ω)*, *φ_ref_(ω)*, and *d* refer to phase difference, the phase of the sample and reference, and the sample thickness, respectively.

According to Fresnel formula, *R_12_*, and *T_12_* respectively mean the reflection and transmission coefficient of the electromagnetic field intensity when the terahertz wave transmits from air to the sample with normal incidence. The *R_12_* and *T_12_* are expressed as the following two equations:

$R_{12}=(ñ_{2}-ñ_{1})/(ñ_{1}+ñ_{2})$ (12)

$T_{12}=2ñ_{1}/(ñ_{1}+ñ_{2})$ (13)

The real part of permittivity(*ε'(ω)*), the imaginary part of permittivity(*ε''(ω)*) and the tangent of dielectric loss ($tan\delta_{\varepsilon}$) can be represented as:

$\varepsilon^{'}\left( \omega\right)=n^{2}\left( \omega\right)-\kappa^{2}(\omega)$ (14)

$\varepsilon^{''}\left( \omega\right)=2\times n\left( \omega\right)\times\kappa(\omega)$ (15)

$\tan\delta_{\varepsilon}=\frac{\varepsilon^{''}\left( \omega\right)}{\varepsilon^{'}\left( \omega\right)}$ (16)

**Detailed analysis of the morphology and electromagnetic properties of films prepared by different LM-deposition methods:**

To investigate the regulation law of preparation processes on the electromagnetic properties of the films, the effects of two different LM-deposition methods on their morphology and electromagnetic response were comparatively studied. The LM was dissolved in an octadecanethiol/ethanol solution for dispersion, and then mixed with the rGO/Fe_2_O_3_ solution. In this case, LM exhibited the characteristic of uniform dispersion as tiny droplets without obvious agglomeration (Figure S11). This structure caused the droplets to fail to form strong reflection of electromagnetic waves like agglomerated LM under the unstretched state. Instead, they only absorbed and scattered electromagnetic waves through polarization and other interactions with graphene sheets, achieving weak electromagnetic shielding with a shielding effectiveness of merely 19.0 dB (Figure S12).

In contrast, directly adding LM without dispersion would cause it to agglomerate, during transmission, electrons encountered relatively small resistance and could conduct more easily inside the LM, thus effectively shielding the external electric field. After stretching, the transmittance change trends of the films prepared by the two deposition methods also showed significant differences: for the film with dispersed LM, the transmittance initially continued rising. Owing to the absence of agglomerates to be pulled apart in the dispersed small droplets, the variation range of electromagnetic wave transmission channels was narrow, with the maximum transmittance only fluctuating between 20% and 50%. When the film was stretched further, although the transmittance started decreasing, the electrical conductivity did not sharply increase, unlike it did in the E-skin fabricated using the directly mixed LM.

The core reason for the significant performance differences caused by different LM-deposition methods is that the LM droplets after dispersion treatment are small in size and uniformly distributed. Even if the oxide layer ruptured during stretching, the effective conductive contact area remained relatively limited. It was difficult to form numerous new effective conductive paths like agglomerated LM, resulting in only a slight increase in conductivity and EMI SE_T_ rising merely to 31.0 dB. Table S1 comprehensively compares the differences in morphological characteristics (LM droplet size, areal density) and shielding performance of the films under different LM contents and deposition conditions, intuitively presenting the regulation law of preparation processes on electromagnetic properties.

**Supplementary Figures**


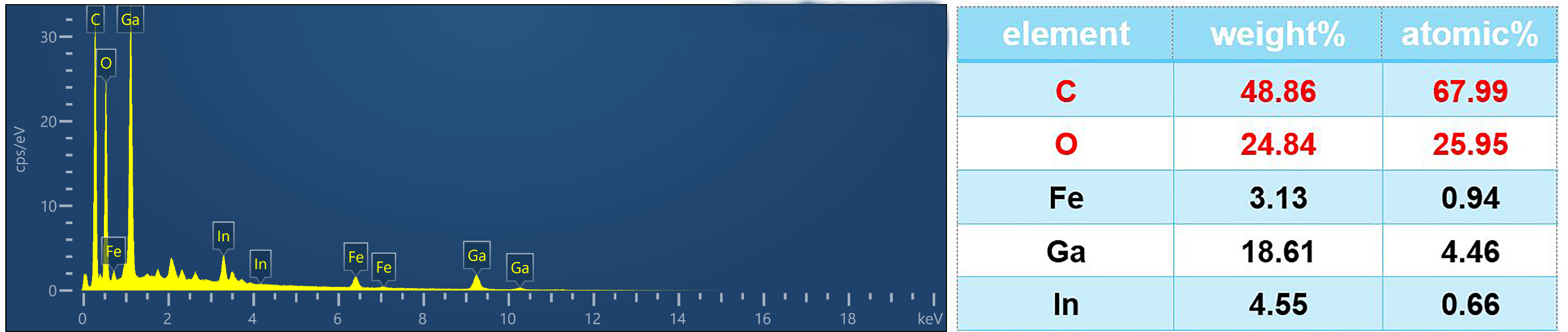


**Figure S1.** EDS mapping of the RATS-E-skin, showing the uniform distribution of C, O, Fe, Ga, and In element.


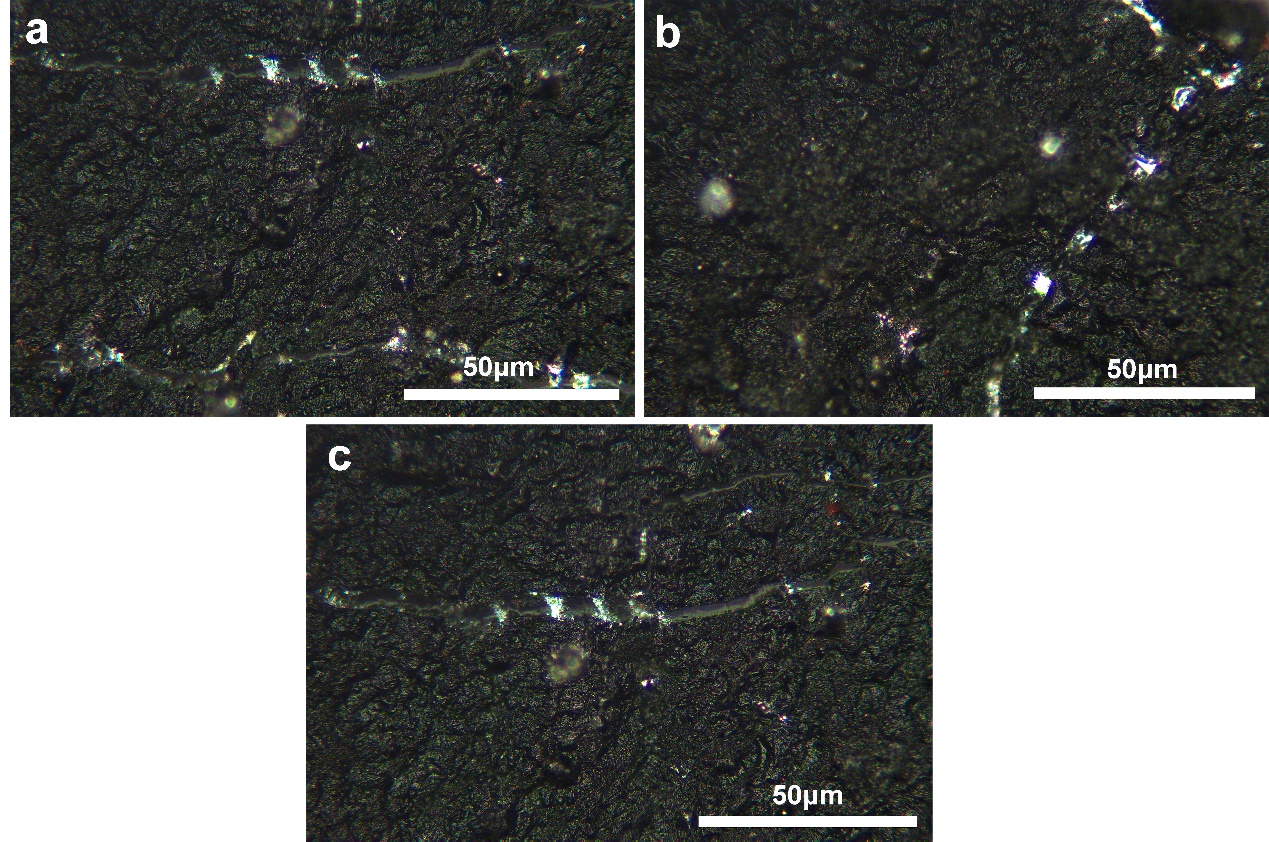


**Figure S2.** Optical microscopy images of the RATS-E-skin under unstretched. The liquid metal is mostly filled in the overlap between the graphene sheets and sheets in the form of small droplets that are dispersed independently, connecting the tightly connected graphene sheets into a conductive pathway.


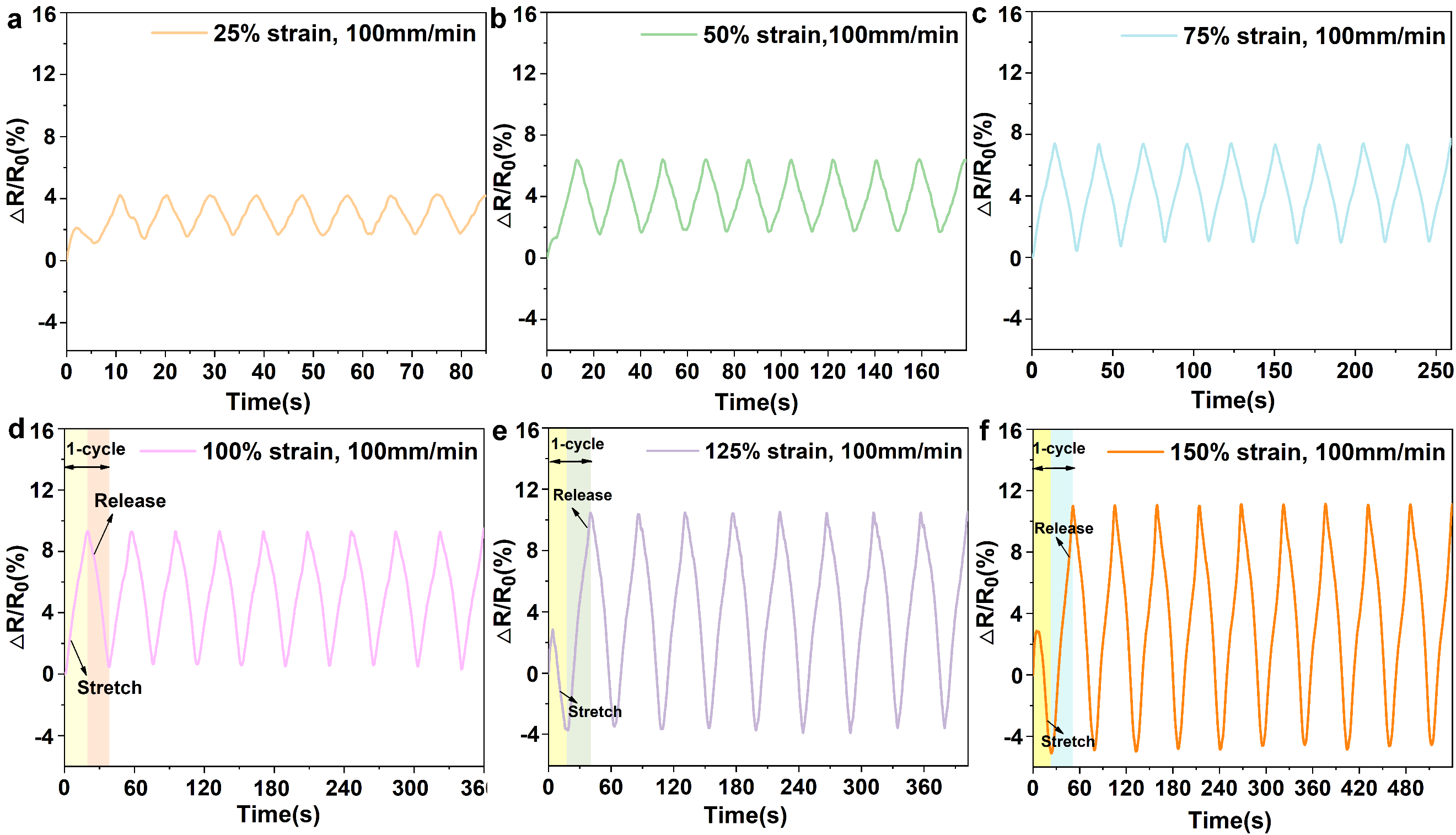


**Figure S3.** Relative resistance changes in the RATS-E-skin during cyclic stretching at a tensile rate of 100mm/min under different strains. (a) 25%, (b) 50%, (c) 75%, (d) 100%, (e) 125%, and (f) 150%.


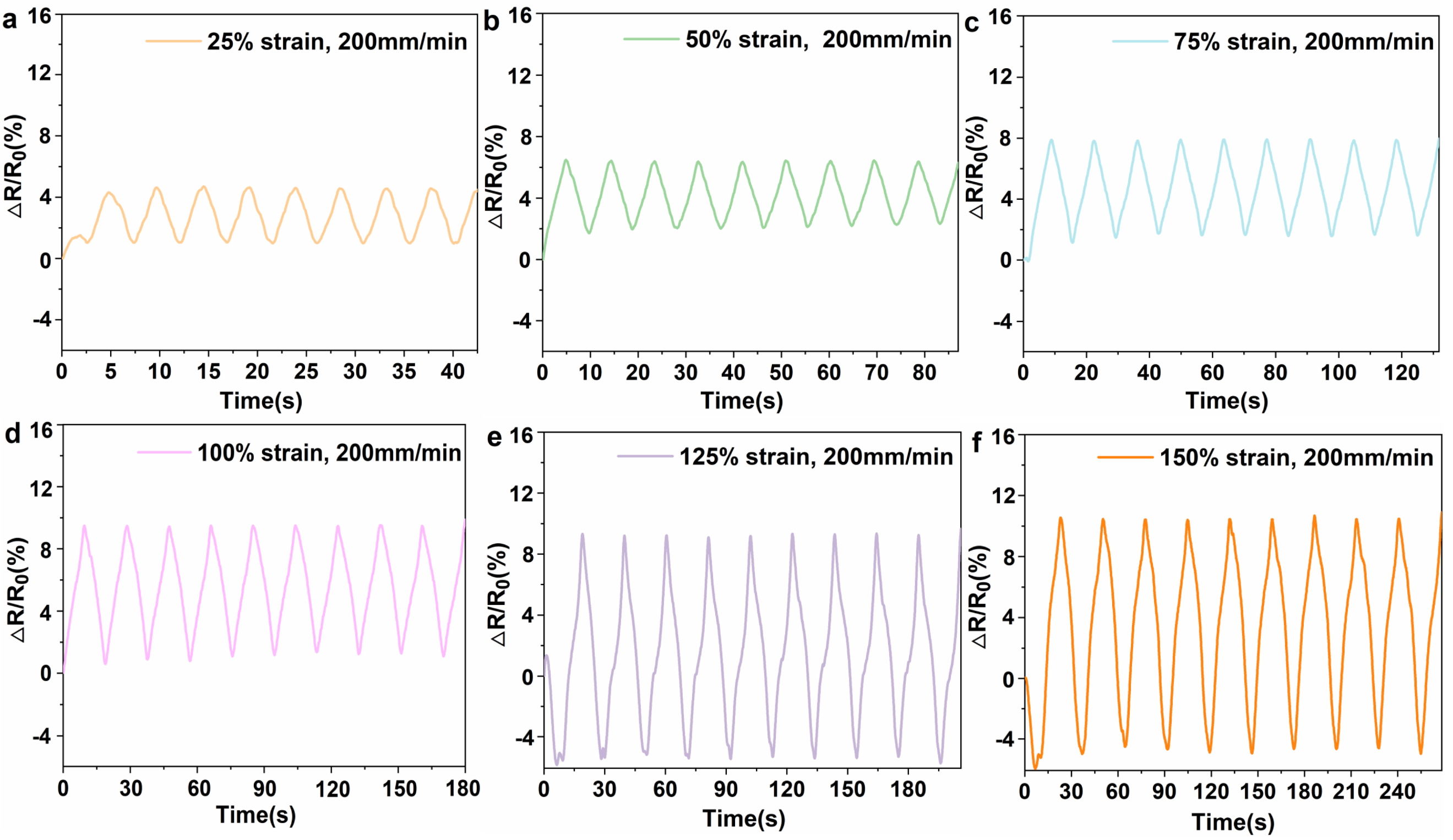


**Figure S4.** Relative resistance changes in the RATS-E-skin during cyclic stretching at a tensile rate of 200mm/min under different strains. (a) 25%, (b) 50%, (c) 75%, (d) 100%, (e) 125%, and (f) 150%.





**Figure S5.** Resistance responses of the RATS-E-skin under different tensile strains, with a tensile rate of 100mm/min.

**
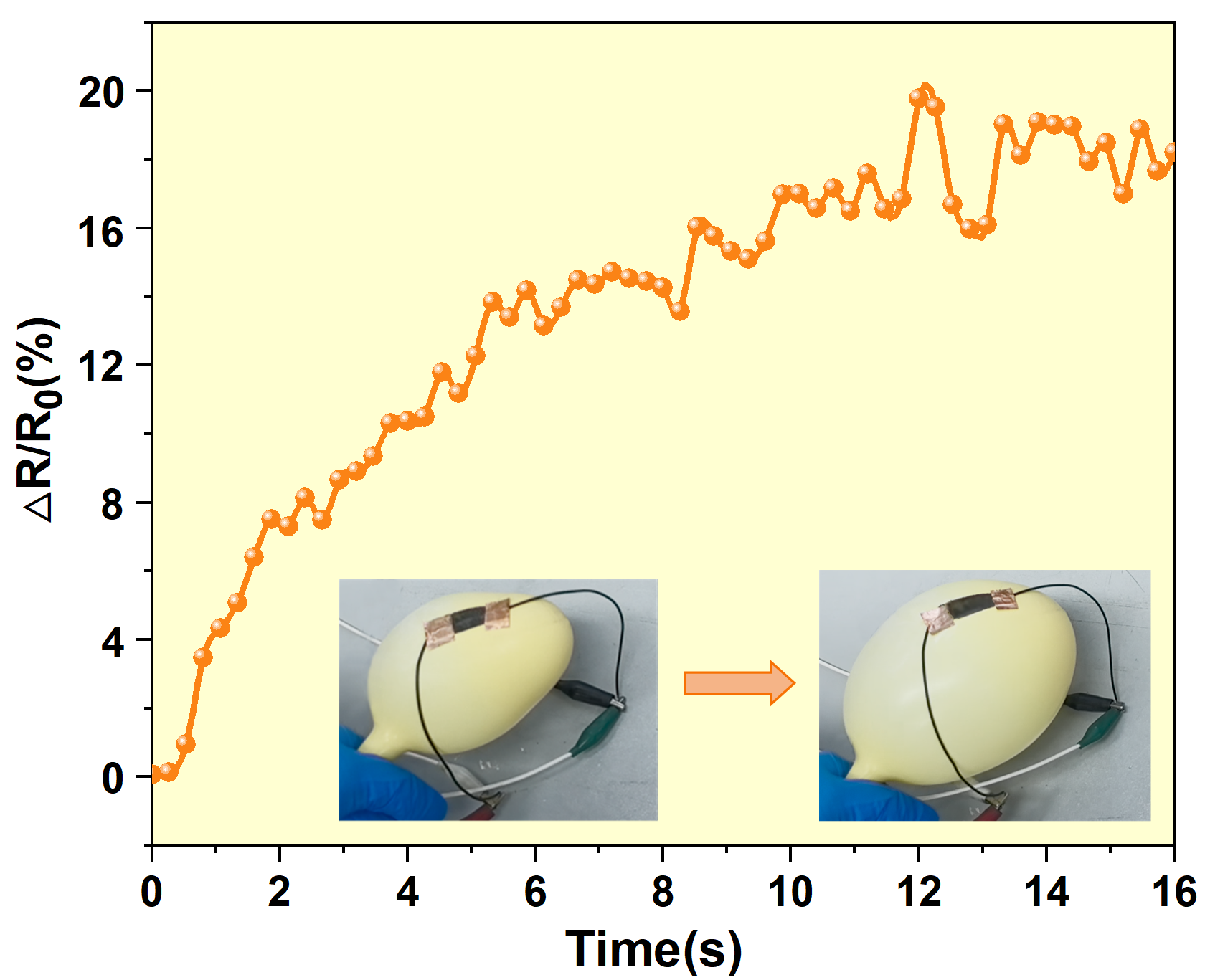
**

**Figure S6.** Relative resistance change of the RATS-E-skin under tensile strain when attached to the balloon surface, simulating the deformation process of deformable equipment.


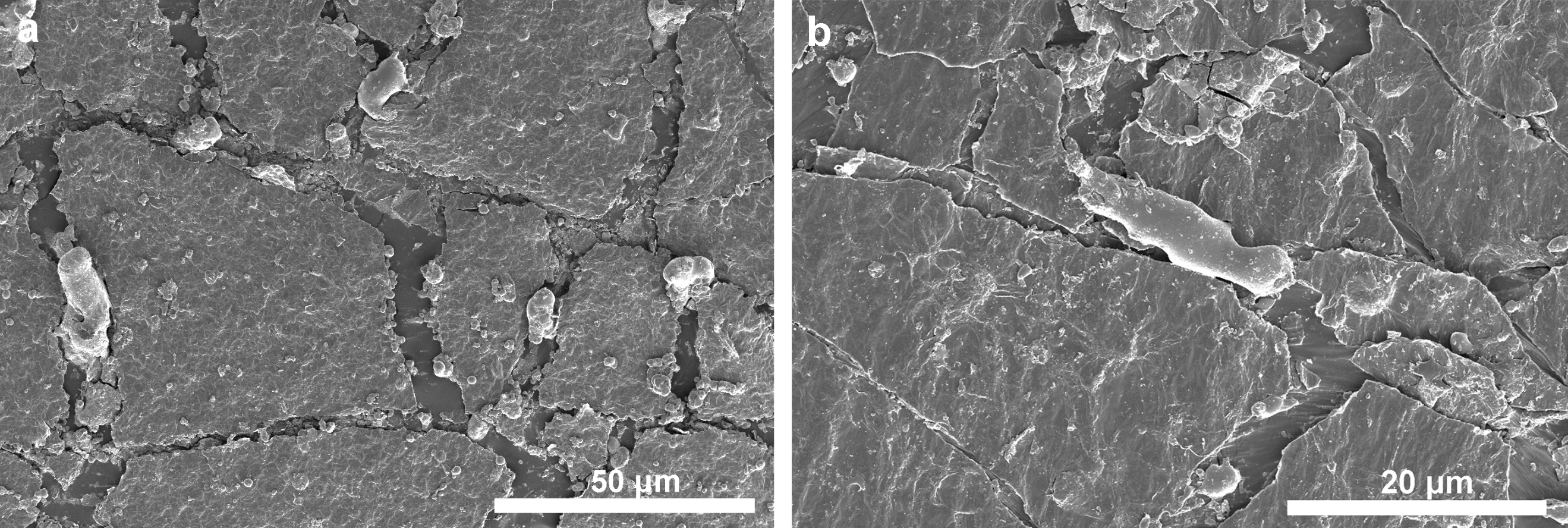


**Figure S7.** SEM images of the RATS-E-skin at tensile strains of 150% show that the LM stretches into metallic wires and reconstructs the conductive path.





**Figure S8.** EDS mapping diagram of the RATS-E-skin in the recovered state after stretching.





**Figure S9.** Terahertz performance curves of the rGO/Fe_2_O_3_/LM film prepared using an LM–rGO ratio of 3:1 under different tensile strains. (a) Transmittance, (b) EMI SE_T_.


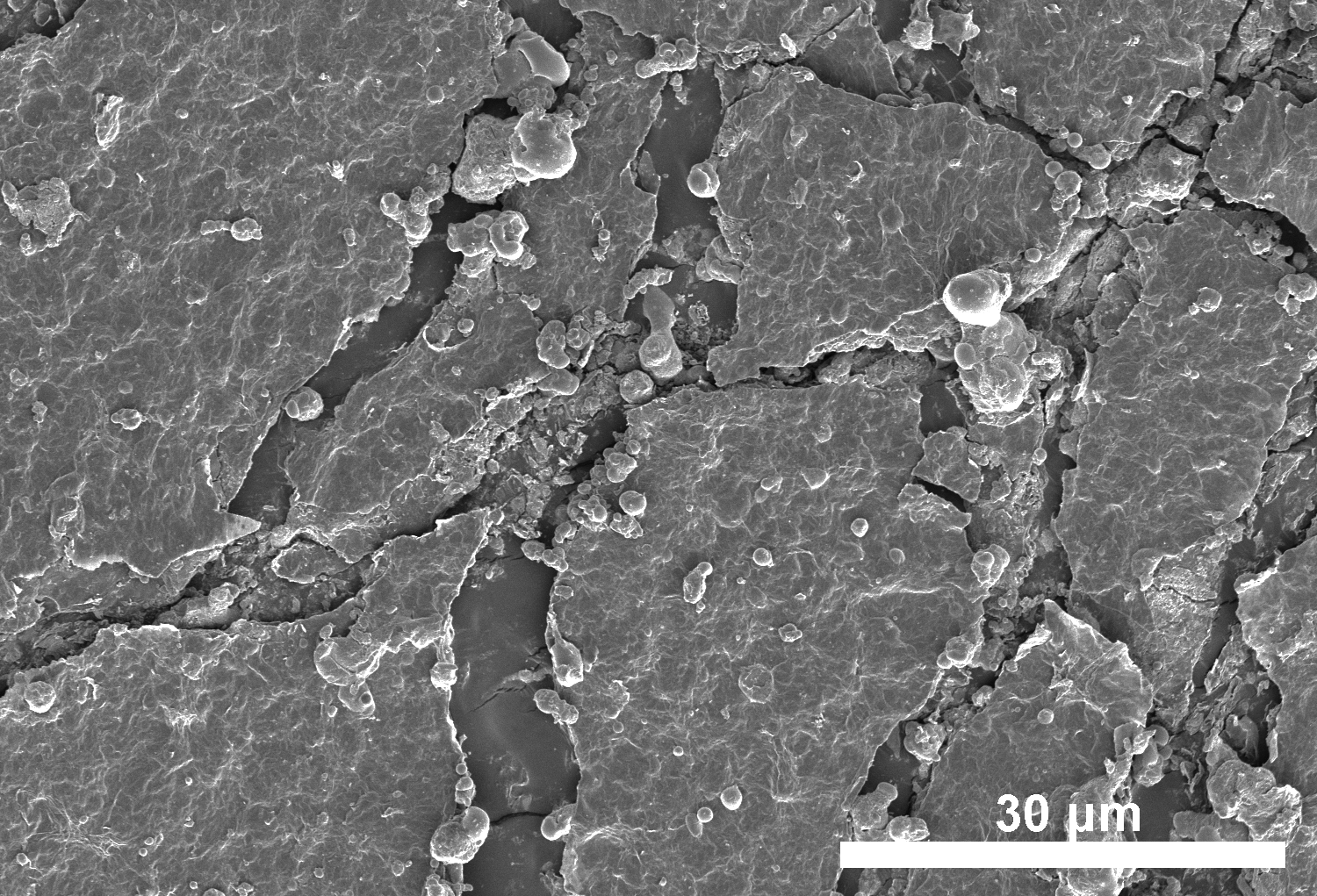


**Figure S10.** SEM images of the rGO/Fe_2_O_3_/LM film prepared with directly added LM using an LM–rGO ratio of 3:1 at tensile strains of 150%.


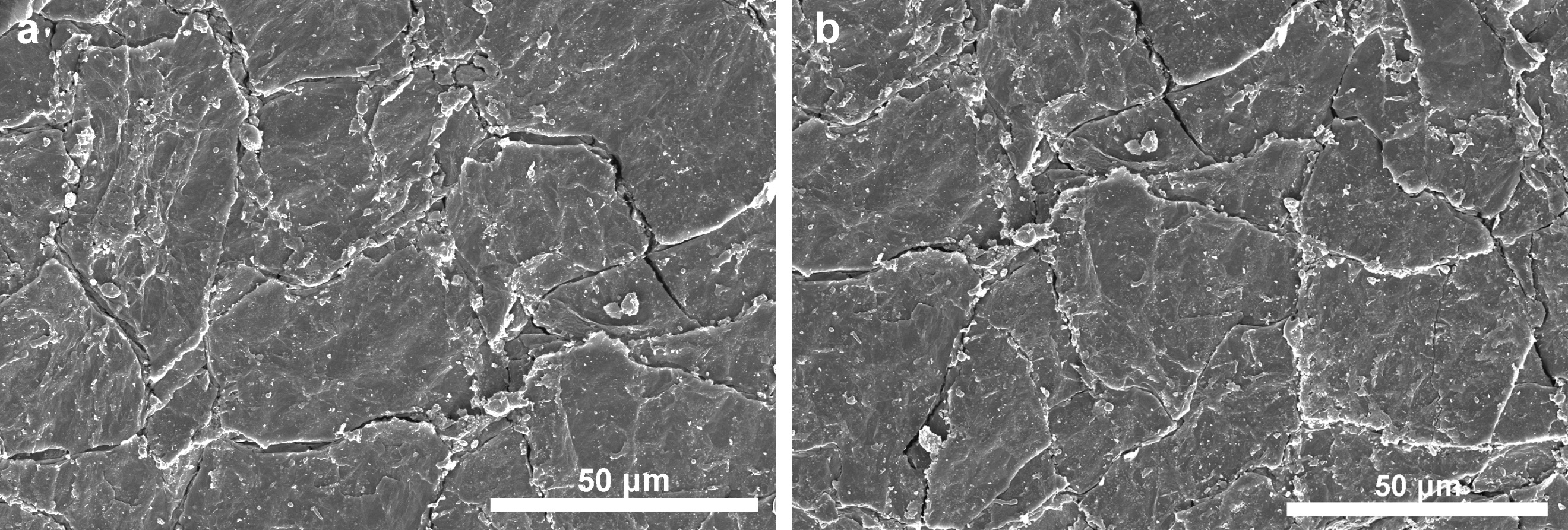


**Figure S11.** SEM images of the rGO/Fe_2_O_3_/LM film with dispersed LM under unstretched.





**Figure S12.** Terahertz performance curves of the rGO/Fe_2_O_3_/LM film with dispersed LM under different tensile strains. (a) Transmittance, (b) EMI SE_T_.


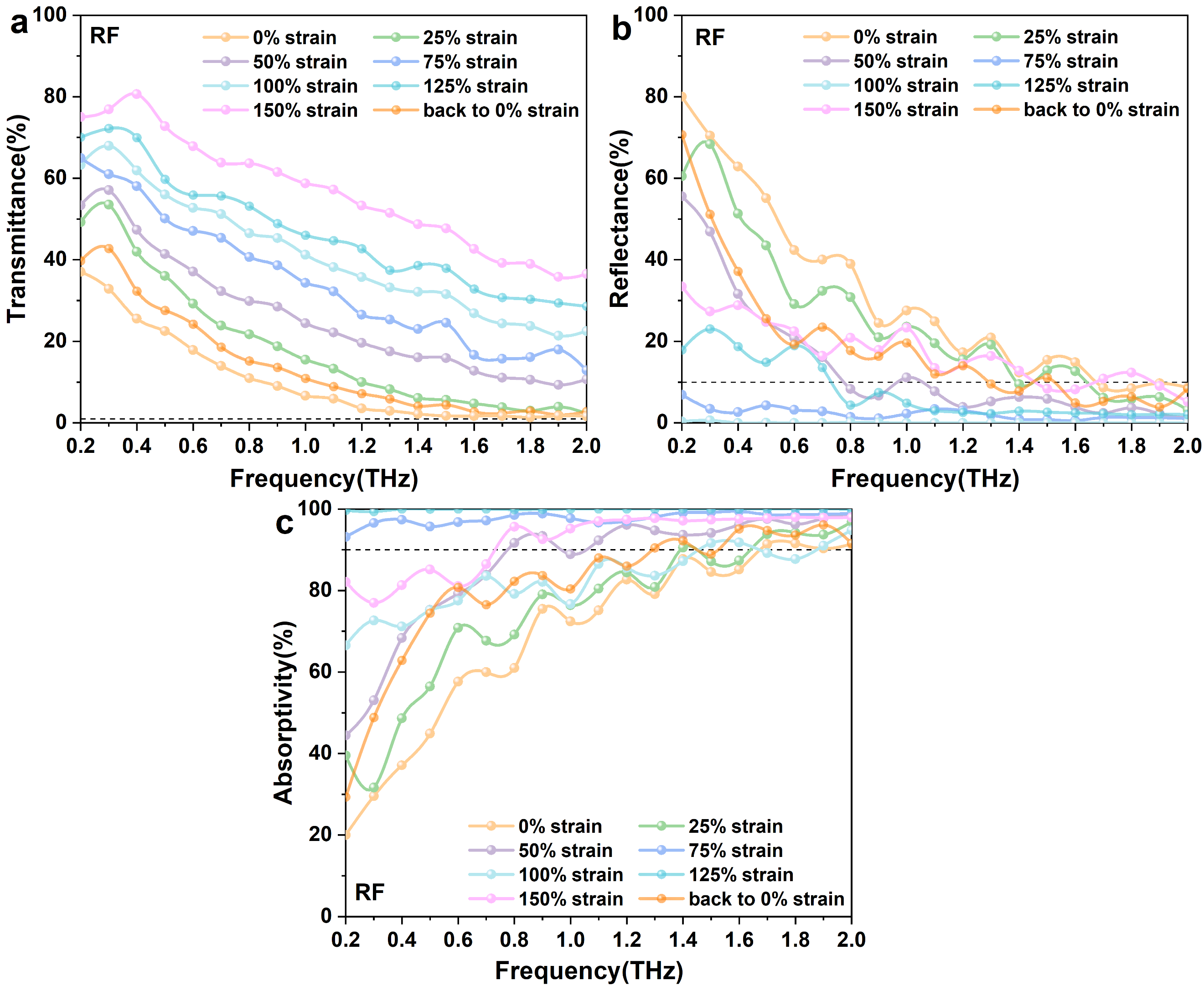


**Figure S13.** Terahertz performance curves of the LM-free RF under different tensile strains. (a) Transmittance, (b) reflectance, and (c) absorptivity.





**Figure S14.** Terahertz performance curves of the pure LM film under different tensile strains. (a) Transmittance, (b) EMI SE_T_, (c) reflectance, and (d) reflection loss.


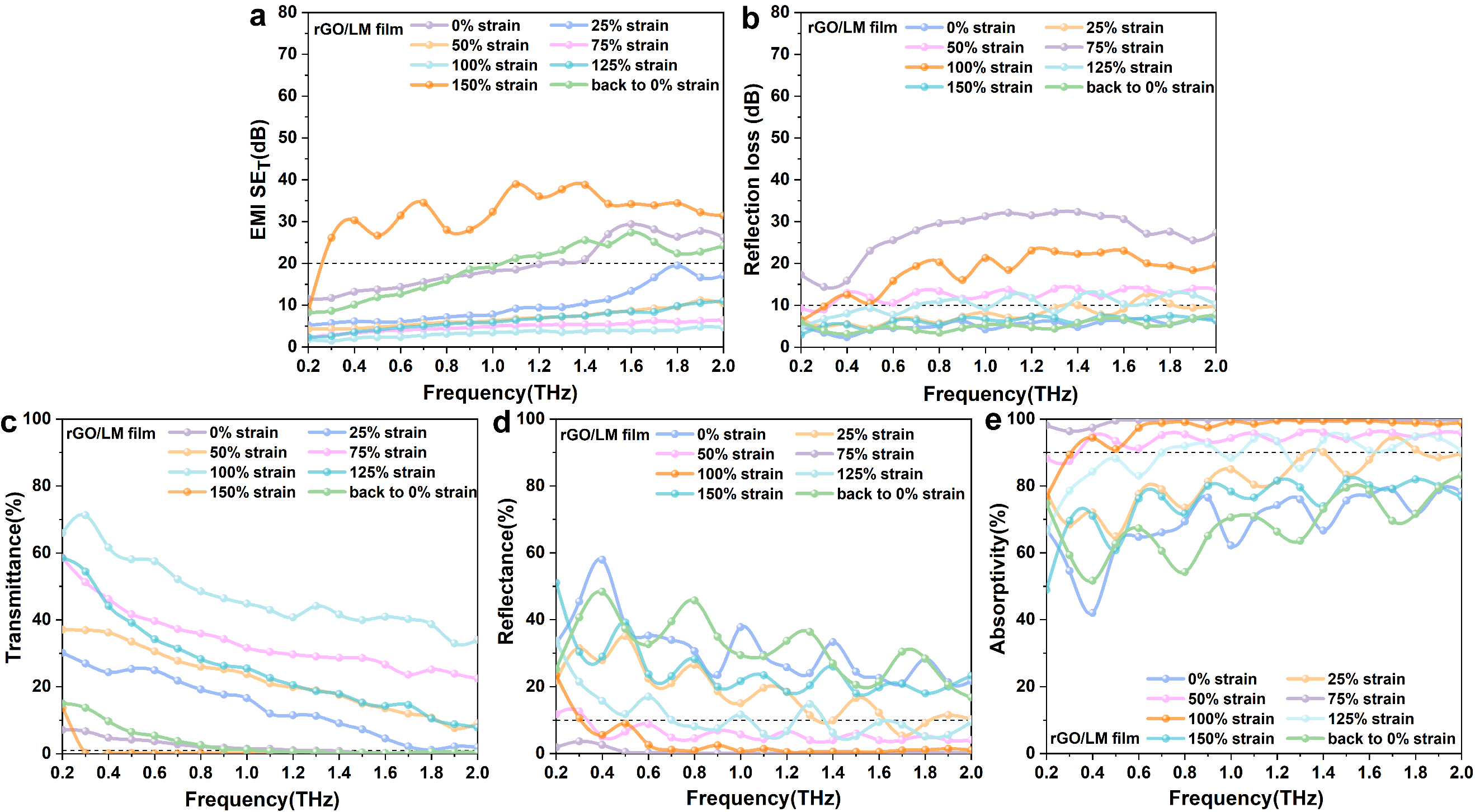


**Figure S15.** Terahertz performance curves the rGO/LM film under different tensile strains. (a) EMI SE_T_, (b) reflection loss, (c) transmittance, (d) reflectance, and (e) absorptivity.


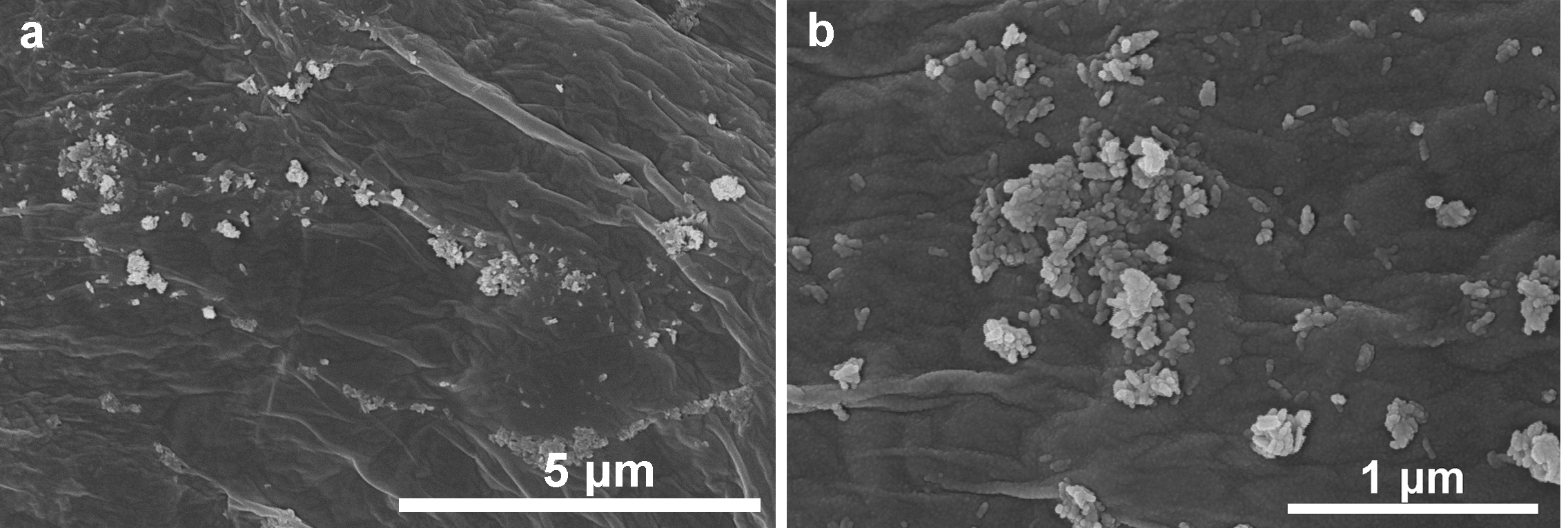


**Figure S16.** SEM images of Fe_2_O_3_ nanorods in the RATS-E-skin under unstretched.





**Figure S17.** Terahertz performance curves of the single-layer rGO/Fe_2_O_3_/LM film containing the directly mixed LM and stretched at different tensile strains. (a) Transmittance, (b) EMI SE_T_, (c) reflectance, and (d) reflection loss.





**Figure S18.** Terahertz performance curves of the three-layer rGO/Fe_2_O_3_/LM film containing the directly mixed LM and stretched at different tensile strains. (a) Transmittance, (b) EMI SE_T_, (c) reflectance, and (d) reflection loss.





**Figure S19.** Terahertz performance curves of the four-layer rGO/Fe_2_O_3_/LM film containing the directly mixed LM and stretched at different tensile strains. (a) Transmittance, (b) EMI SE_T_, (c) reflectance, and (d) reflection loss.





**Figure S20.** Transmittance and EMI SE_T_ curves of composite films containing the dispersed LM and stretched at different tensile strains. (a-b) Single-layer, (c-d) three-layer, and (e-f) four-layer rGO/Fe_2_O_3_/LM film.





**Figure S21.** Reflectance and reflection loss curves of LM-free composite films and stretched at different tensile strains. (a-b) Three-layer and (c-d) four-layer rGO/Fe_2_O_3_ film.


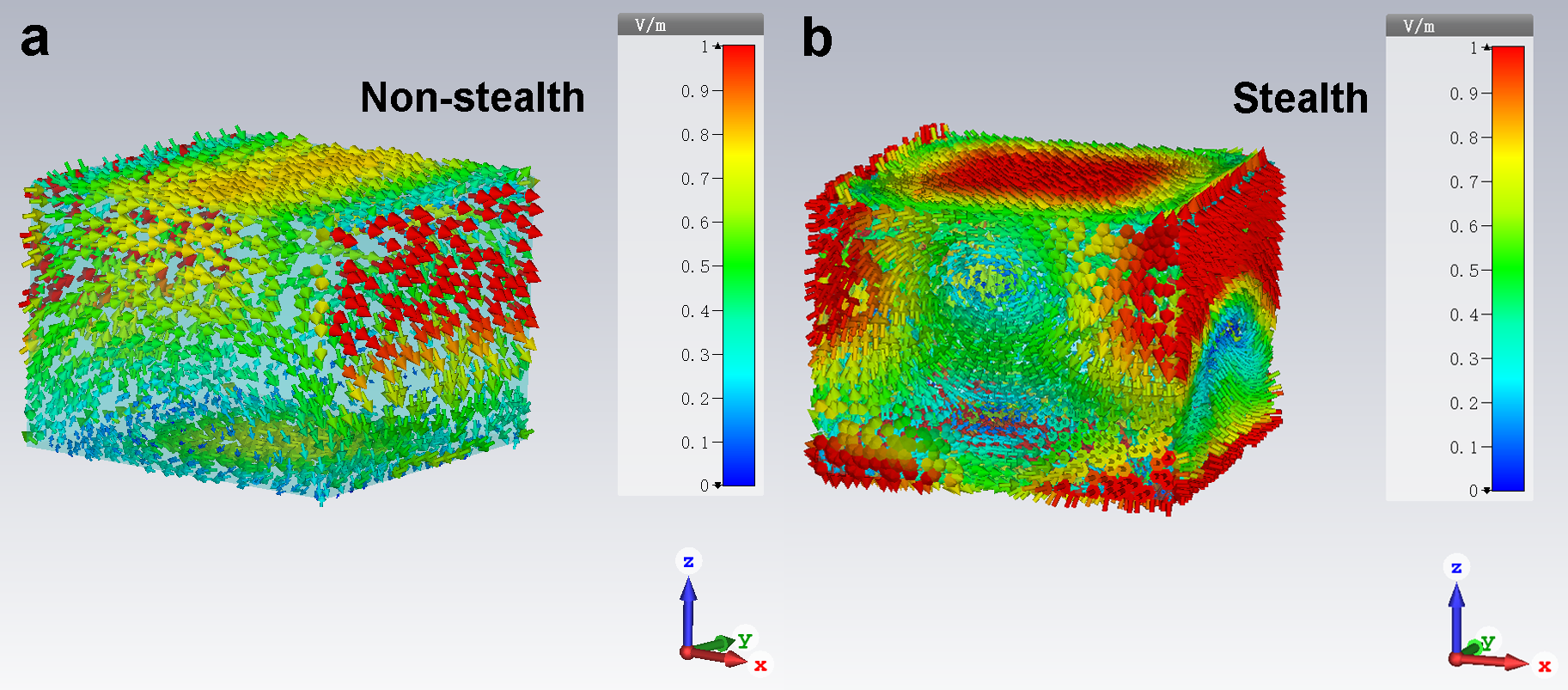


**Figure S22.** Absorption relevant electric field of the RATS-E-skin model. (a) Non-stealth and (b) stealth.


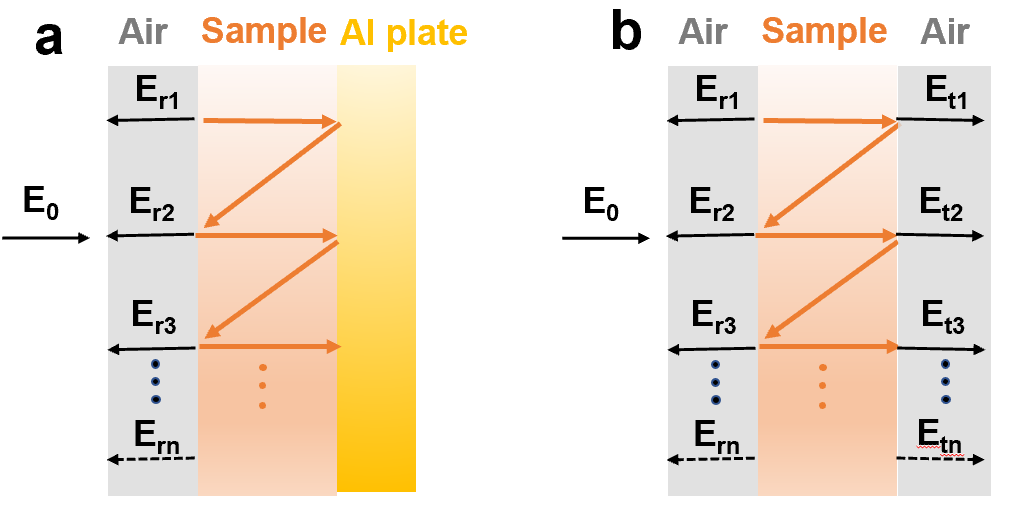


**Figure S23.** Schematic diagram of terahertz model. (a) Absorption and (b) electromagnetic shielding.

**Supplementary Tables**

**Table S1.** Electromagnetic properties comparison of the liquid metal phase in rGO/Fe_2_O_3_/LM films under different deposition and conditioning conditions.

| **Deposition condition** | **Conditioning condition (m_LM_: m_rGO_）** | **LM droplet size and content** | **LM droplet** **distribution characteristics** | **0% strain maximum EMI shielding effectiveness** | **100% strain transmittance range and maximum transmittance** | **150% strain maximum EMI shielding effectiveness** | **Shielding properties switching modes and effective shielding bandwidth (EMI ＞20dB)** |
| --- | --- | --- | --- | --- | --- | --- | --- |
| direct adding of LM | 2：1 | 2-4µm, 0.0537g·cm^-2^ | isolated agglomeration distribution | 32.4 dB | 50%-80%  T_max_=76.7% | 50.8 dB | On(33.3%)-off(0%)-on(91.1%) |
| direct adding of LM | 3：1 | 3-5µm, 0.0767 g·cm^-2^ | dense agglomeration distribution | 36.2 dB | 40%-60%  T_max_=60.8% | 53.2 dB | On(44.4%)-off(0%)-on(88.9%) |
| dispersion of LM | 2：1 | ＜1-2µm, 0.0592g·cm^-2^ | dispersed droplet distribution | 19.0 dB | 20%-50%  T_max_=45.5% | 31.0 dB | Off(0%)-off(0%)-on(12.2%) |

**Table S2.** Comparison of the properties among electromagnetic functional materials in open literature.

| **Materials** | **Minimum reflection loss(dB)** | **Maximum reflection loss(dB)** | **Effective absorption bandwidth(RL＞10dB)** | **Minimum**  **EMI shielding effectiveness (dB)** | **Maximum**  **EMI shielding effectiveness (dB)** | **Effective shielding bandwidth (EMI＞20dB)** | **Refs.** |
| --- | --- | --- | --- | --- | --- | --- | --- |
| **RATS-E-skin** | **4.71** | **60.24** | **1.8THz (100%)** | **3.43** | **50.81** | **1.64THz (91.1%)** | **this work** |
| PEDOT:PSS/cellulose aerogel | N/A | N/A | 1THz(100%) | 0.46 | 16.99 | 0THz | [2] |
| Ti_3_C_2_T_x_ MXene sponge foam | N/A | 65 | 1.35THz (100%) | N/A | 40 | 1.35THz(100%) | [13] |
| Ti_3_C_2_T_x_ film with wrinkled structures | N/A | N/A | N/A | 6.1 | 15.2 | 0THz | [14] |
| PNIPAM/PEDOT: PSS/MXene aerogel | N/A | N/A | N/A | 15.5 | 59.3 | 4.2GHz(100%) | [3] |
| rGO/VO_2_ aerogel | 15 | 64 | 7.27GHz (45.4%) | N/A | N/A | N/A | [4] |
| Functional carbon spring | 5 | 35 | 13.36 GHz (83.5%) | N/A | N/A | N/A | [5] |
| Bionic octopus-inspired C/Fe_3_C foam | 10 | 57.6 | 7.8GHz (48.75%) | N/A | N/A | N/A | [6] |
| GO/QC hydrogel | 10 | 53.99 | 6GHz(37.5%) | N/A | N/A | N/A | [7] |
| PANI/SA hydrogel | N/A | N/A | N/A | 3 | 25.8 | 4.2GHz(100%) | [8] |
| PMCP hydrogel | N/A | N/A | N/A | 9.3 | 53.9 | 4GHz(100%) | [9] |
| M-organohydrogel | N/A | N/A | N/A | 1.3 | 39.3 | 5.6GHz(100%) | [10] |
| Magnetic liquid metal film | N/A | N/A | N/A | 20 | 80 | 4.2GHz(100%) | [11] |
| rGO/PPy/PUF | N/A | N/A | N/A | 7.5 | 40 | 4.2GHz(100%) | [12] |
| VO_2_/PVDF-HFP composite film | N/A | N/A | N/A | 10 | 50 | 10GHz(100%) | [15] |
| Wood-derived lamellar aerogel | N/A | N/A | N/A | 2 | 23 | 4.2GHz(100%) | [16] |
| Graphene foam | 7.6 | 33.2 | 50.5GHz (80.9%) | N/A | N/A | N/A | [17] |
| CNF/GO aerogel | 15.2 | 46.1 | 6.8GHz (42.5%) | N/A | N/A | N/A | [18] |
| Printing of Al^3+^-MXene inks | N/A | N/A | N/A | 8.2 | 34 | 4.2GHz(100%) | [19] |

**References**

[1] Huang, Z.; Chen, H.; Ma, W.; Huang, Y.; Zhu, D.; Chen, Y. Research Progress on Terahertz Stealth and Shielding Materials. *Chem. J. Chinese Universities*. **2019**, *40*, 1103–1115.

[2] Kuang, C.; Chen, S.; Luo, M.; Zhang, Q.; Sun, X.; Han, S.; Wang, Q.; Stanishev, V.; Darakchieva, V.; Crispin, R.; Fahlman, M.; Zhao, D.; Wen, Q.; Jonsson, M. P. Switchable Broadband Terahertz Absorbers Based on Conducting Polymer-Cellulose Aerogels. *Adv. Sci.* **2024**, *11*, 2305898.

[3] Yan, Q.; Liu Z.; Xiong, J.; Lian, H.; Chen, H.; Fei, T.; Chen, Y.; Zheng, H.; Zhao, X.; Xu, L.; Xue, F.; Zhong, Y.; Ma, X.; Shi, L.; Peng, Q.; He, X. Stimuli-Responsive MXene/PNIPAM Hydrogel WITH High-Performance and Tunable Electromagnetic Interference Shielding Performance. *Adv. Sci.* **2025**, *12*, e05551.

[4] Cheng, Z.; Wang, R.; Cao, Y.; Cai, Z.; Zhang, Z.; Huang, Y. Intelligent Off/On Switchable Microwave Absorption Performance of Reduced Graphene Oxide/VO2 Composite Aerogel. *Adv. Funct. Mater.* **2022**, *32*, 2205160.

[5] Wang, Z. Y.; Li, Z. C.; Li, B.; Shi, A. F.; Zhang, L.; Zhu, Y. B.; Ye, F.; Yu, S. H. Functional Carbon Springs Enabled Dynamic Tunable Microwave Absorption and Thermal Insulation. *Adv. Mater.* **2024**, 2412605.

[6] Fang, G.; He, T.; Hu, X.; Yang, X.; Zheng, S.; Xu, G.; Liu, C. Bionic Octopus Structure Inspired Stress-Driven Reconfigurable Microwave Absorption and Multifunctional Compatibility in Infrared Stealth and De-Icing. *Chem. Eng. J.* **2023**, *467*, 143266.

[7] Chen, P.; He, S.; Zou, Z.; Wang, T.; Hu, J.; Tao, J.; Yang, L. Intelligent Hydrogels Enabled Large-Scale Variability in Continuously Tunable Microwave Absorption. *Adv. Funct. Mater.* **2025**, 2506308.

[8] Wang, Y. Q.; Cao, M.; Liu, B. W.; Zeng, F. R.; Fu, Q.; Zhao, H. B.; Wang, Y. Z. Controllable Proton-Reservoir Ordered Gel towards Reversible Switching and Reliable Electromagnetic Interference Shielding. *Mater. Horizons* **2023**, *11*, 978–987.

[9] Tang, J.; Gao, Y.; Li, T.; Qin, R.; Qi, Q.; Meng, F. Thermoresistive Network in Phase-Transition Hydrogel: Achieving on/off Switchable Electromagnetic Interference Shielding. Adv. Funct. Mater. **2025**, *2504959*.

[10] Fang, M.; Huang, L.; Cui, Z.; Yi, P.; Zou, H.; Li, X.; Deng, G.; Chen, C.; Geng, Z.; He, J.; Sun, X.; Shui, J.; Yu, R.; Liu, X. Phase-Transition Microcapacitor Network in Organohydrogel for Absorption-Dominated Electromagnetic Interference Shielding and Multi-Mode Intelligent Responsiveness. *Adv. Funct. Mater.* **2024**, 2418870.

[11] Zhu, R.; Li, Z.; Deng, G.; Yu, Y.; Shui, J.; Yu, R.; Pan, C.; Liu, X. Anisotropic Magnetic Liquid Metal Film for Wearable Wireless Electromagnetic Sensing and Smart Electromagnetic Interference Shielding. *Nano Energy* **2022**, *92*, 106700.

[12] Zhang, X.; Sun, Q.; Liang, X.; Gu, P.; Hu, Z.; Yang, X.; Liu, M.; Sun, Z.; Huang, J.; Wu, G.; Zu, G. Stretchable and Negative-Poisson-Ratio Porous Metamaterials. *Nat. Commun.* **2024**, *15*, 392.

[13] Shui, W.; Li, J.; Wang, H.; Xing, Y.; Li, Y.; Yang, Q.; Xiao, X.; Wen, Q.; Zhang, H. Ti3C2Tx MXene Sponge Composite as Broadband Terahertz Absorber. *Adv. Opt. Mater.* **2020**, *8*, 2001120.

[14] Yang, S.; Lin, Z.; Wang, X.; Huang, J.; Yang, R.; Chen, Z.; Jia, Y.; Zeng, Z.; Cao, Z.; Zhu, H.; Hu, Y.; Li, E.; Chen, H.; Wang, T.; Deng, S.; Gui, X. Stretchable, Transparent, and Ultra-Broadband Terahertz Shielding Thin Films Based on Wrinkled MXene Architectures. *Nano-Micro Lett.* **2024**, *16*, 165.

[15] Liang, S.; Guan, H.; Zhang, H.; Han, X.; Zhao, J.; Dou, S.; Hao, S.; Zhou, H.; Geng, C.; Zhao, T.; Gu, J.; Wei, H.; Li, Y. Tunable High-Performance Electromagnetic Interference Shielding of VO2 Nanowires-Based Composite. *ACS Appl. Mater. Interfaces* **2024**, *16*, 21024−21033.

[16] Liu, X.; Li, Y.; Sun, X.; Tang, W.; Deng, G.; Liu, Y.; Song, Z.; Yu, Y.; Yu, R.; Dai, L.; Shui, J. Off/on Switchable Smart Electromagnetic Interference Shielding Aerogel. *Matter* **2021**, *4*, 1735–1747.

[17] Zhang, Y.; Huang, Y.; Zhang, T.; Chang, H.; Xiao, P.; Chen, H.; Huang, Z.; Chen, Y. Broadband and Tunable High-Performance Microwave Absorption of an Ultralight and Highly Compressible Graphene Foam. *Adv. Mater.* **2015**, *27*, 2049–2053.

[18] Kang, S.; Qiao, S.; Cao, Y.; Hu, Z.; Yu, J.; Wang, Y. Compression Strain-Dependent Tubular Carbon Nanofibers/Graphene Aerogel Absorber with Ultrabroad Absorption Band. *Chem. Eng. J.* **2022**, *433*, 133619.

[19] Li, L.; Qi, C. Z.; Chen, M.; He, P.; Min, P.; Zhou, X.; Yu, Z. Z.; Zhang, H. Bin. High-Precision Printing of Flexible MXene Patterns for Dynamically Tunable Electromagnetic Interference Shielding Performance. *ACS Appl. Mater. Interfaces* **2024**, *16*, 13082–13090.
